# Physical activity barriers and facilitators in patients awaiting spine surgery: A qualitative study

**DOI:** 10.1371/journal.pone.0355173

**Published:** 2026-08-04

**Authors:** José Manuel García-Moreno, Stephan U. Dombrowski, Dresden Forshner, Alex Lui, Erin Bigney, Jillian Kearney, Neil Manson, Christopher Small, Niels Wedderkopp, Jeffrey J. Hébert

**Affiliations:** 1 Faculty of Kinesiology, University of New Brunswick, Fredericton, New Brunswick, Canada; 2 Canada East Spine Centre, Saint John, New Brunswick, Canada; 3 UNB Saint John, Saint John, New Brunswick, Canada; 4 Horizon Health Network, Saint John, New Brunswick, Canada; 5 UNB Fredericton, Fredericton, New Brunswick, Canada; 6 Sant John Orthopaedics, Saint John, New Brunswick, Canada; 7 Dalhousie University Faculty of Medicine, Saint John, New Brunswick, Canada; 8 Division of Orthopaedic Surgery, Horizon Health Network, Saint John, New Brunswick, Canada; 9 Center for Research in Childhood Health, University of Southern Denmark, Odense, Denmark; 10 School of Allied Health, Murdoch University, Perth, Australia; Soochow University, CHINA

## Abstract

**Background:**

Degenerative spine conditions and physical inactivity are major public health concerns. Engaging in physical activity before spine surgery may improve postoperative outcomes experienced by patients with lumbar stenosis or lumbar disc herniation. This study aims to identify barriers and facilitators to physical activity among patients with degenerative lumbar spine conditions awaiting surgery.

**Methods:**

In this qualitative patient-oriented study, guided by a patient partner who contributed to the development of the interview guide and interpretation of findings, we conducted data-prompted semi-structured interviews (a method in which personalised physical activity information is used to stimulate reflection and discussion), with patients diagnosed with lumbar spinal stenosis or lumbar disc herniation on a surgical waiting list. Interviews were structured around the Theoretical Domains Framework (TDF). Patient responses were deductively coded to TDF domains and inductively analyzed to identify subthemes. Key domains were determined through researcher consensus based on the frequency and clarity of specific beliefs within each domain.

**Results:**

We interviewed 18 patients (8 female), 11 with lumbar spinal stenosis and 7 with lumbar disc herniation, with a mean (SD) age of 54.7 (17.3) years. Five key TDF domains were identified: skills, beliefs about capabilities, beliefs about consequences, environmental context and resources, and emotion. Barriers to physical activity included a lack of physical capacity, pain and other adverse symptoms, potential for negative consequences, inappropriate environment, and negative emotions. Facilitators to physical activity were motivation, appropriate environment, supportive social influences, positive emotions, alternate strategies, and daily physical activity habits.

**Conclusions:**

Physical activity behaviour in patients awaiting spine surgery is shaped by an interplay of physical, emotional and contextual factors. These findings can inform the design of tailored, theory-driven preoperative physical activity interventions, targeting key barriers such as pain, fear of harm, and limited access to resources, while leveraging factors including motivation, professional guidance, social support, and habit formation.

## Introduction

Degenerative spine conditions [1] and physical inactivity [2] are major public health concerns. Low back pain and related disorders remain one of the leading causes of disability worldwide [3]. Lumbar spinal stenosis and lumbar disc herniation are among the most common degenerative spinal conditions contributing to this burden, and both are associated with substantial pain, disability, and reduced quality of life. Lumbar spinal stenosis affects one in five older adults [4,5], and is characterised by functional limitations (e.g., reduced walking capacity due to neurogenic claudication), leg pain, back pain, and numbness or weakness in the lower extremities [6]. Similarly, disc herniation typically presents with back pain accompanied by lower extremity pain, tingling, numbness, or weakness [7–9].

While spinal surgery can offer substantial relief for many patients, about one in three patients with lumbar stenosis [10] and one in four patients with disc herniation [11] experience relatively poor surgical outcomes [10]. Persistent spinal pain type 2 is associated with greater disability than stroke and heart failure [12], and carries a significant economic burden [13]. Although physical inactivity is acknowledged as a leading cause of death, morbidity, and socioeconomic burden [14], engaging in physical activity before spinal surgery may also improve postoperative outcomes experienced by patients with lumbar stenosis [15] or lumbar disc herniation [16].

Previous qualitative research has explored the preoperative experiences of patients with lumbar stenosis or lumbar disc herniation who participate in structured exercise-based programs [17], but little is known about patients’ broader views on physical activity in every day life before surgery. Limited qualitative research has examined the behavioural determinants of general physical activity engagement (as opposed to structured, supervised exercise) in patients awaiting lumbar spine surgery using a theory-informed framework. This distinction is clinically meaningful, as barriers and facilitators to general physical activity may differ substantially from those encountered in structured, supervised settings. Only 17% of patients meet the WHO-recommended levels of physical activity while waiting for lumbar spine surgery [18]. Understanding the factors that may influence physical activity behaviour in this population is a critical first step toward developing effective interventions. To systematically identify these determinants, the Theoretical Domains Framework (TDF) was used. The TDF is a validated synthesis of 33 behaviour change theories organised into 12 domains, designed to provide a comprehensive and structured approach to understanding the factors that influence health-related behaviours [19,20]. Compared to broader behavioural models such as the Capability, Opportunity, Motivation-Behaviour (COM-B) model [21], the TDF offers greater granularity, enabling a more detailed exploration of the cognitive, emotional, and contextual factors that may influence behaviour. Furthermore, the TDF has been specifically validated for use in qualitative research aimed at identifying barriers and facilitators to behaviour change [20], and has been applied in patients with musculoskeletal and other chronic conditions [22–24].

Accordingly, this study aimed to identify potential preoperative behavioural determinants (barriers and facilitators) to engagement in physical activity among surgical candidates with lumbar stenosis or lumbar disc herniation, using a theory-informed qualitative framework.

## Methods

A qualitative study design was chosen as the most appropriate approach to address the research question. While survey-based approaches can quantify the prevalence of barriers and facilitators, they are constrained by predefined response options and may fail to capture the full range and depth of patients’ experiences and perspectives [25]. A qualitative approach, by contrast, allows patients to describe their physical activity behaviour in their own words, revealing nuanced cognitive, emotional, and contextual factors that structured instruments are unlikely to detect [26]. This is particularly relevant in a surgical population where the lived experience of pain, functional limitation, and uncertainty about surgery is likely to shape physical activity behaviour in ways that cannot be fully anticipated in advance. The use of the TDF as a guiding framework further ensured that the qualitative data were collected and analyzed in a systematic and theoretically grounded manner [20].

### Patients

Eligible patients were identified from the Canadian Spine Outcomes and Research Network (CSORN) registry, all from the Saint John Regional Hospital, a tertiary care center. Inclusion criteria were: adult surgical candidates (≥19 years) with lumbar spinal stenosis or lumbar disc herniation who had not previously undergone lumbar spinal surgery. A randomised contact order was generated using an online randomization tool, and patients were contacted sequentially in that order to avoid selection bias. Of the 87 patients identified in the registry, 64 met the eligibility criteria, as some had already undergone surgery between registry entry and the time of contact. Of these, 18 consented to participate. The remaining 46 patients did not participate for the following reasons: 23 were not interested in the study, 19 did not respond to the call, and 4 were unable to complete the online interview or could not travel to the interview location. No systematic data were collected on non-participating patients, and therefore a formal comparison between participants and non-participants was not possible.

This study protocol was approved by the Research Ethics Boards from the Horizon Health Network (2024−3370) and the University of New Brunswick (2024−109). All patients provided signed informed consent, and the study was conducted in accordance with the Declaration of Helsinki.

### Data sources

Demographic and clinical variables were obtained from the CSORN database. Back and leg pain intensity were assessed with separate 0–10 Numeric Rating Scales [27] with a similar scale implemented to quantify leg tingling, burning, and numbness. Pain-related disability was assessed with the modified Oswestry Disability Index (mODI), with scores ranging from 0 to 100 [28]. Health-related quality of life was evaluated using the EuroQol Visual Analogue Scale (EQ-VAS), with scores ranging from 0 to 100 [29].

### Interview topic guide

We conducted Data-Prompted Interviews, a qualitative method in which personalised data are used during the interview to stimulate reflection and discussion [30]. In this study, the data prompts were based on each patient’s self-reported physical activity behaviour, collected at the beginning of the interview, thereby helping patients to recall, interpret, and elaborate on their experiences. Interviews were structured based on the TDF [19,20], which has been used to identify factors influencing physical activity in different settings, including patients following coronary artery bypass graft surgery [23] and heart failure [24].

To ensure consistent identification of barriers and facilitators in this population, we developed a semi-structured interview topic guide based on the TDF (see S1 File). Each patient participated in a single, individual interview. The topic guide included open-ended questions tailored to each patient’s personalised physical activity data, with additional prompts to explore each theoretical domain [30]. The target behaviour was defined as “the things you do that contribute to achieving 150 minutes of moderate-to-vigorous physical activity per week”, in line with the Canadian 24-Hour Movement Guidelines for Adults [31].

The topic guide was developed through consensus among several researchers (E.B., S.D., J.G., J.H.) and a patient representative (A.L.), a person with lived experience of degenerative lumbar spine conditions. A.L. contributed to the development of the interview topic guide by providing feedback on the relevance and acceptability of the questions from a patient perspective, participated in the consensus meetings during the analysis phase, reviewed the categorisation of interview extracts into the primary domains confirming their alignment with the patient perspective, reviewed the final themes upon completion of the analysis, and presented the preliminary results at a conference. The topic guide was piloted in two mock interviews to ensure clarity, understanding of the questions, and effective use of the prompts. Additionally, to stimulate discussion and enhance understanding, an A4 sized prompt card featuring images of different physical activity categories was explained to patients and displayed during the interview (see S2 File). Interviews were designed to be patient-centred, allowing for the modification of questions throughout the study to improve both the clarity and overall experience for the patients.

### Procedure

Patients were recruited between 21 August 2024 and 12 February 2025. Interviews were conducted between the same period. All patients provided written informed consent. The interviewers received training from an experienced qualitative researcher with expertise in health behaviour change and data-prompted interviews (S.D.). The training included simulated interviews followed by a pilot interview with a patient representative. All interviews were conducted by two male co-investigators (D.F., J.G.). D.F. was an undergraduate student researcher with no prior experience in qualitative research but with experience in clinical research involving this patient population. J.G. was an experienced physiotherapist and post-doctoral researcher with clinical and research expertise in patients with degenerative lumbar spine conditions. Neither interviewer was involved in the clinical care of the included patients, minimizing the potential for bias or assumptions related to prior clinical responsibilities. Prior to data collection, the interviewers were made aware of how their professional backgrounds and assumptions might influence in their interviews and interpretation of findings, as part of their training with S.D. Specifically, J.G.’s background as a physiotherapist with clinical expertise in degenerative lumbar spine conditions may have led to assumptions about the barriers and facilitators patients experience, potentially influencing the formulation of follow-up questions during interviews and the interpretation of response during coding. To mitigate this, reflexivity was actively encouraged throughout the research process: field notes were used to document assumptions during interviews, coding decisions were made under the supervision of S.D. and discussed until consensus was reached, and the use of the TDF as a predefined theoretical framework helped to structure the analysis and limit the influence of subjective interpretation. All interviews were conducted either in consultation rooms or online, based on patient preference, and were audio recorded, transcribed verbatim and anonymised. Field notes were taken to record emotional responses and non-verbal communication, and were also drawn upon during the analytical process to provide additional context. Prior to the interview, patients were informed that the interviewers were members of the research team with an academic interest in understanding barriers and facilitators to physical activity among patients awaiting spine surgery. No interviews were repeated. Patients received a modest honorarium for their participation.

During the interview, patients first completed the Godin-Shephard Leisure-Time Physical Activity Questionnaire, a brief, three-item questionnaire to assess their weekly exercise behaviours. They were asked, “During a typical 7-day period (a week), how many times on average do you engage in the following types of exercise for more than 15 minutes during your free time?” The exercise categories offered were “strenuous exercise”, “moderate exercise”, and “mild/light exercise”. Patients provided numerical responses for each category. Based on their score, patients were classified as “active” if they accumulated 24 points or more, “moderately active” if they accumulated 14–23 points, and “insufficiently active/sedentary” if they accumulated fewer than 14 points [32]. The questionnaire results were then used as a data-prompt to contextualise and initiate the discussion, helping patients reflect on their activity level relative to the recommended target of 150 minutes of moderate-to-vigorous physical activity per week. The second part of the interview involved applying the topic guide, with questions and prompts adapted to each patient. Patients were informed of their right to access and review their transcriptions to verify, validate, and make any necessary corrections; however, no requests were made.

Although qualitative studies do not require a predetermined sample size, a final sample of 18 patients is consistent with empirical evidence indicating that saturation is typically achieved within 9–17 interviews in qualitative studies with relatively homogeneous populations and narrowly defined objectives [33]. Data saturation was determined following the approach recommended by Francis et al [34]: after an initial set of 10 interviews were conducted and analyzed, three additional interviews were conducted and analyzed to assess whether new barriers or facilitators emerged. If new themes were identified, three further interviews were conducted and analyzed. This iterative process was repeated until no new themes emerged across three consecutive interviews, at which point data saturation was considered reached. Recruitment and analysis were conducted simultaneously to allow ongoing saturation assessment. Consensus regarding data saturation was achieved among three researchers (S.D., J.G., J.H.). The Consolidated Criteria for Reporting Qualitative Research (COREQ) were used to ensure that all aspects of the qualitative research were reported [35] (see S3 File).

### Analysis

Transcriptions were stored as digital documents and analyzed manually, without the use of software. Coding was conducted line-by-line directly within the interview transcripts. In line with published TDF guidance [20], the use of qualitative data analysis software is not mandated; analytical rigour derives from the systematic and transparent application of the analytic framework rather than the tools used to organise data. Data from the interviews were analyzed using the 2005 version of the TDF [19], which comprises 12 domains: knowledge, skills, social/professional role and identity, beliefs about capabilities, beliefs about consequences, motivation and goals, memory, attention and decision processes, environmental context and resources, social influences, emotion, behavioural regulation, and the nature of behaviours. Qualitative data analysis was conducted using a hybrid deductive/inductive approach, following established recommendations for TDF-based qualitative research [20] involving the following steps:

1. Interviews were read multiple times by J.G. to ensure familiarity with the data.
2. The first two interviews were manually coded by J.G. under the supervision of S.D. to develop a shared coding strategy and resolve initial uncertainties. A content analysis approach was used to allocate responses deductively to relevant theoretical domains (multiple-domain allocation was possible).
3. The remaining interviews were manually coded by J.G., consulting S.D. whenever uncertainty arose regarding domain allocation. Coding was performed by a single researcher (J.G.). In all cases of uncertainty, consensus was reached through discussion between J.G. and S.D. This supervised approach and ongoing consensus process are consistent with established recommendations for TDF-based qualitative research [20], where coding decisions are guided by a predefined theoretical framework and consensus-based resolution of disagreements is considered an appropriate alternative to independent dual coding [36].
4. Data saturation was reached, as the final three transcripts revealed no additional barriers or facilitators beyond those already identified.
5. Summaries of domain codings were inductively grouped into subthemes by J.G. and reviewed in an initial meeting with S.D., J.H., and J.G. A second meeting was then held with E.B., A.L., D.F., J.H., and J.G., using the outcomes of the first meeting to further refine and finalise the subthemes. The patient representative (A.L.) participated in this process to ensure both the categorisation of interview extracts into the primary domains and the resulting subthemes aligned with the patient perspective.
6. Relevant theoretical domains were identified through consensus discussions among the aforementioned co-investigators (J.G., D.F., S.D., A.L., E.B., J.H.). Domain relevance was determined based on the following criteria when the domain acted as a barrier: (i) high frequency of specific beliefs, assessed by comparing the number of coded quotes across domains (see S1 Table), and (ii) presence of clear beliefs, defined as statements that were explicitly and unambiguously related to physical activity behaviour and expressed consistently across multiple patients. Frequency was used alongside belief clarity and consistency, in line with recommendations that domain relevance in TDF-based research should reflect the convergence of multiple indicators rather than quote count alone [20]. The (iii) existence of conflicting beliefs was also documented for information but was not used as a criterion for relevance. Facilitators were documented and reported within each key domain but were not used as criteria for determining domain relevance. The criteria used to classify TDF domains as key barriers are summarised in S4 File, which also provides additional details on domains that functioned as facilitators. Interviewed patients were not asked to provide feedback on the study findings.

## Results

A total of 18 patients (8 female, 10 male) consented to participate. Among these, 11 had lumbar stenosis and 7 had lumbar disc herniation. Demographic and clinical characteristics are presented in Table 1.

**Table 1:** Patient information.

	Total n = 18	Lumbar Stenosis n = 11	Lumbar Disc Herniation n = 7
Demographic variables			
Female sex	8	4	4
Age (years; mean ± SD)	54.7 ± 17.2	60.8 ± 15.3	45 ± 18.0
Marital status			
Married/engaged/common-law	12	7	5
Single	4	2	2
Divorced/separated	1	1	0
Widowed	1	1	0
Living status			
Living with a Partner/roomate/family	15	9	6
Living alone	3	2	1
Highest education level			
High school diploma	6	4	2
Technical school or associate degree	4	3	1
College/university degree/undergraduate degree	6	3	3
Post graduate or professional degree	2	1	1
Working status			
Currently working	6	3	3
Employed but not working	2	0	2
Retired	10	8	2
Interview mode (online/in person)	7/11	4/7	3/4
Clinical variables			
BMI (mean ± SD)	31.8 ± 7.1	33.7 ± 6.0	29.1 ± 8.0
Back pain intensity (0–10) (mean ± SD)	6.3 ± 2.5	6.2 ± 2.1	6.6 ± 3.2
Leg pain intensity (0–10) (mean ± SD)	6.2 ± 2.4	5.8 ± 2.5	6.7 ± 2.4
Leg tingling/burning/numbness intensity 0–10 (mean ± SD)	6.4 ± 1.9	6.7 ± 2.0	5.8 ± 1.7
mODI (0–100) (mean ± SD)	43.4 ± 12.4	40.2 ± 8.1	48.6 ± 15.8
Overall health status from 0 to 100 (mean ± SD)	55.8 ± 21.0	52.3 ± 17.6	61.4 ± 24.3
GSLTPAQ category			
Insuffiently active/sedentary	7	5	2
Moderately active	1	0	1
Active	10	6	4
Number of days in waiting list for surgery, from the decision to operate to interview (mean ± SD)	244.3 ± 186.4	183.3 ± 75.4	344.1 ± 249.9

BMI: body mass index; EuroQol: self-rated quality of life; GSLTPAQ: Godin-Shephard Leisure-Time Physical Activity Questionnaire; mODI: modified Oswestry Disability Index; SD: standard deviation.

The 18 interviews were performed in person and online (11 in person and 7 online), and the duration ranged from 18 to 48 minutes, with a mean of 35.5 minutes (SD = 8.2). Data saturation was assessed when the interview 18 finished and the stop was decided.

### Key and other domains

Domains were classified as key or other to prioritise those areas with the strongest perceived influence on physical activity behaviour in this population, which may inform both clinical practice and the development of future interventions. The criteria used to classify domains as key are described in the Methods section and summarised in S4 File. All 12 theoretical domains were identified. Within each domain, specific subthemes were identified and described as barriers, facilitators, or both, reflecting the context-dependent nature of each factor. Illustrative quotes are marked with (b) for barriers and (f) for facilitators.

#### 1. Key domains.

Five domains were categorised as key: *skills; beliefs about capabilities; beliefs about consequences; environmental context and resources;* and *emotion.* Across the five key domains, pain and functional limitation emerged as recurring transversal themes, shaping patients’ capabilities, consequences, emotions, and contextual circumstances simultaneously. Rather than operating in isolation, these factors coexisted and reinforced one another, creating a complex interplay that influenced physical activity behaviour. To avoid redundancy, pain and functional limitation are described in depth within the domain where they were most prominently expressed, namely beliefs about capabilities, and their connections with other domains are highlighted where relevant.

**Skills:** Two subthemes emerged: *injury hinders physical activity* and *guidance and instructions for physical activity.*

The subtheme *injury hinders physical activity* represented a barrier, as the lumbar condition limited patients’ ability to engage in physical activity. Many found physical activity ‘*kind of difficult’* or *‘very difficult’*, citing limitations in strength, range of motion, endurance, and balance. These physical limitations reflect both the direct impact of pain and associated symptoms on bodily function (as described in depth under beliefs about capabilities) and the deconditioning that results from prolonged inactivity driven by those symptoms. Balance issues were particularly frequent, with patients reporting that balance *‘isn’t great’, ‘seems off’*, or *‘is wonky’*.

“I can’t trust my legs and hips to do anything that is [a] big exertion” (b) (Patient 4, 70–79 years, lumbar stenosis)

The subtheme *guidance and instructions for physical activity* was also closely linked to pain and its consequences. Patients reported that professional guidance was necessary not only due to unfamiliarity with physical activity, but primarily because of the need to move safely in the context of their symptoms. This caution mirrors the fear of harm described under beliefs about consequences, where avoidance of pain exacerbation shaped behavioural decisions. Lack of guidance was a barrier, with patients saying they needed someone to *‘show me how to do those properly’*, while those who received it described it as a facilitator, noting that it *‘taught me a lot’,* and *‘was very helpful’.*

“I wouldn’t really know where to start in a gym, or how to lift or where to lift; and I would be a little bit timid about my back and, like, not having any instruction.” (b) (Patient 1, 30–39 years, lumbar disc herniation)

“It was good to have guidance to make sure I was doing it right, they [the physiotherapists] were getting the most benefit whatever I was doing.” (f) (Patient 14, 70–79 years, lumbar stenosis)

**Beliefs about capabilities:** Two subthemes were identified: *pain severity and associated symptoms,* and *comorbidities.*

Patients’ main barriers were *pain severity and associated symptoms,* including intense back pain, leg pain, and leg tingling, burning, or numbness. Symptoms were often described as overwhelming or unpredictable, referred to as *‘loss of sensation’, ‘muscle pain’, ‘tightness’,* and *‘pins and needles’*. No meaningful differences in perceived capability were identified between patients with lumbar stenosis and those with lumbar disc herniation, nor were differences related to age or time on the waiting list apparent, reflecting the chronic and pervasive nature of both conditions. Notably, patients responded differently to similar levels of pain: some attempted to push through discomfort and maintain activity, while others ceased activity immediately upon symptom onset. These individual differences suggest that beliefs about capability are shaped not only by objective physical limitations but also by personal thresholds and coping styles. In contrast, milder symptoms facilitated physical activity, with patients feeling *‘confident’* or *‘in control’* when performing physical activity *‘at a modified level’.*

“The only problem is that I had constant pain ever since I injured my back the first time, and it’s been constant.” (b) (Patient 7, 60–69 years, lumbar stenosis)

“When I deal with those pins and needles, and that numbness, and stuff like that, like, I can push through it if needed.” (f) (Patient 9, 30–39 years, lumbar stenosis)

Many patients reported *comorbidities*, either age-related or associated with their spinal condition and physical inactivity, including anemia, blood pressure problems, arthritis, and urinary incontinence. These additional health issues further compounded patients’ perceived incapacity, reinforcing a cycle in which reduced activity worsened comorbidities, which in turn further limited physical activity.

“Besides running around with my grandson when I can, I really don’t [do any physical activity at the moment]. I don’t have a lot [physical activity] because I’m in pain. I’ve been in pain now for a decade, so yeah, not necessarily the back thing, just like I say, I’m full of arthritis and my joints are all messed up.” (b) (Patient 10, 60–69 years, lumbar stenosis)

**Beliefs about consequences:** Four subthemes were identified: *improving overall health and well-being, avoidance of negative consequences, modification of behaviour,* and *concern of long-term consequences*.

*Improving overall health and well-being* was a common facilitator for physical activity*.* Patients mentioned benefits like *‘mental health’, ‘longevity’, ‘sleep better’, ‘lose weight’,* and *‘strength’*, though notably only one patient identified a direct back-related benefit, noting that *‘the more I stretch, the less discomfort I feel’.* This suggests that patients perceived physical activity as beneficial for general health but struggled to connect it specifically to their spinal condition, which may partly explain why fear of harm often outweighed perceived benefits. No patient reported any benefit from avoiding physical activity; instead, inactivity was seen as being linked to *‘health deteriorate’, ‘fatness’, and ‘depression’.*

“I think the results of doing physical activity are better physical shape, […] healthier heart, cardiovascular […], losing weight, […], adding years to my life, hopefully.” (f) (Patient 9, 30–39 years, lumbar stenosis)

*Avoidance of negative consequences* was a prominent barrier, rooted not in abstract concern but in concrete prior experiences of symptom exacerbation following physical activity. Patients had learned through experience that certain activities reliably triggered or worsened their symptoms, leading to a rational but limiting pattern of avoidance. Patients expressed a need to *‘not take too many risks’, ‘be cautious’,* considered physical activity ‘*not a smart thing’*, or felt they *‘would just be choosing to hurt myself’*.

“It […] prevents me from doing stuff the way I’d like to, as far as exercising […]. Or if I’m helping somebody, you know, if somebody asks you for help in a physical manner to lift something […], you kind of try to limit yourself so you don’t hurt yourself, basically.” (b) (Patient 13, 50–59 years, lumbar disc herniation)

The *modification of behaviour* to minimise pain or harm was common and acted as a barrier, as it aimed to protect patients by reducing levels of physical activity. This experiential learning drove patients to *‘try to judge by my body’s reaction’, ‘listen to my body’,* or *‘hear from my back’* during physical activity.

“I have to be careful with what I’m doing and how I’m moving. And, you know, there is a lot of pain, so, it’s like, I’m cautious in the gym. What exercises that I’m doing.” (b) (Patient 5, 40–49 years, lumbar stenosis)

Patients expressed *concern of long-term consequences*, which acted as a barrier. They believed physical activity often caused delayed negative effects, even without immediate discomfort, anticipating symptom onset hours or days after activity. Together, these subthemes reflect a self-protective behavioural pattern in which the memory of past pain episodes became a powerful deterrent to engagement. After physical activity, patients felt they were *‘gonna pay for it’,* or *‘shouldn’t have done that’*, often referring back or leg symptoms.

“I know if I do something […], I’ll pay for it [in] the morning. Well, tonight I’ll feel it.” (b) (Patient 15, 50–59 years, lumbar disc herniation)

**Environmental context and resources:** Five subthemes were identified: *build and natural environment, access to private and financial resources, household and work responsibilities, access to healthcare system and health professionals,* and *weather.*

The subtheme *build and natural environment,* such as parks, walkable roads, and natural settings like trails, acted as facilitators. Patients with access to these environments described how being in nature *‘drives you to go for some exercise’*, especially when *‘roads are clear to walk’*, referring to lakes, trails, nature parks, or beaches. Accessibility was key, as *‘proximity makes it very easy’*. In contrast, limited or unsafe access acted as a barrier, with patients emphasizing the need for terrain to be *‘safely’* walkable and free of *‘risk slipping or falling’*, a concern that for many was amplified by the balance difficulties and functional limitations described under skills.

“I’m in apartment, so sometimes I find that difficult to be outside and do yoga, or do exercises of any kind outside because it’s not really my yard, It’s my landlords’ yard. They live upstairs, and being in an apartment, it kind of makes you feel like you have to leave to exercise, like, you have to go outside your apartment because it’s a small space.” (b) (Patient 1, 30–39 years, lumbar disc herniation)

“I live in a small town that has a lot of nature, and it’s also pretty [safe because of] the people who live there. They are pretty used to see runners, so it’s quite safe. I feel very safe running. So, it’s just easy to go out your front door, like, sometimes I might drive somewhere to some trails, or I can just run from my house to the trails.” (f) (Patient 8, 30–39 years, lumbar disc herniation)

*Access to private and financial resources*, such as a home or workplace gym, or assistive tools like walking sticks, facilitated physical activity by making it more accessible. In contrast, some patients reported financial difficulties as barriers, limiting access to gyms or physical activity programs, which they described as *‘pricey’, ‘terribly expensive’*, or lacking financial support, as in *‘don’t have coverage’*.

“Now, I feel like a financial burden because there is nothing that supports you when you are waiting to have surgery.” (b) (Patient 12, 30–39 years, lumbar disc herniation)

“I was in my own house […] in a room where there’s a big, huge TV, and I felt part of that [chair exercise] class, and I just thought […] I had won the lottery when I found it on YouTube.” (f) (Patient 6, 60–69 years, lumbar stenosis)

*Household and work responsibilities* acted as facilitators when they involved physical effort, such as walking, climbing stairs, or manual labor, with some patients reporting that daily duties constituted their main source of physical activity, such as *‘the walking I get is running my errands’,* or jobs with *‘a lot of stairs and walking’*. Conversely, these responsibilities acted as barriers when work demands or family obligations limited time or energy for additional physical activity.

“If work is super busy, I wouldn’t get in [physical activity].” (b) (Patient 5, 40–49 years, lumbar stenosis)

“I go up and down the stairs many times a day because I live in a basement […] at least six times a day […] seven days [a week]” (f) (Patient 12, 30–39 years, lumbar disc herniation)

*Access to the healthcare system and health professionals* acted as a facilitator for some patients, supporting engagement in physical activity through resources such as a *‘home program of exercises’,* an *‘online training type of program’,* or a *‘book full of every exercise’*. This support was particularly valuable given patients’ need for safe, guided exercise in the context of their pain and functional limitations, as highlighted under skills. In contrast, limited access acted as a barrier for those who lacked a family doctor or did not know how to apply for rehabilitation programs.

“I don’t really have a family doctor.” (b) (Patient 5, 40–49 years, lumbar stenosis)

“I do physical education here at the hospital, [where] you can have physiotherapy, but anyway, there was a waiting list at the time when I first needed it. And I had gone to (Name of the doctor), he recommended that, […] and then (Name of another doctor) […] met several of us up in (Name of a place), and it was sort of like an open seminar, and he spoke with us.” (f) (Patient 3, 70–79 years, lumbar disc herniation)

*Weather*, such as, rain, snow, and cold, acted as a barrier by making it difficult, unpleasant, or unsafe to perform physical activity outdoors. For patients already managing balance difficulties and fear of fall linked to their lumbar condition, adverse weather conditions further heightened the perceived risk of injury, compounding the concerns described under beliefs about consequences. Patients described it as *‘just the snow and the really cold, that’s my deterrent’*, or said, *‘If it’s 20 below, I don’t want to leave the house’.* In contrast, warmer *weather* acted as a facilitator by encouraging outdoor physical activity and making it more comfortable and appealing.

“Yes, […] it’s crap. If it’s really cold, I don’t wanna go out. I can go out in the rain and I go out […] in nice weather or whatever. But if it is really cold, I’m not going out because first of all, it’s gonna seize everything, and nobody wants to be out there freezing their face off.” (b) (Patient 12, 30–39, lumbar disc herniation)

“If I could [do physical activity], today the weather is really nice. So, I really wanna be outside today. So, like, it’s a big motivating factor for me. […] If it’s nice [out], [I] try to be outside at least.” (f) (Patient 2, 30–39 years, lumbar disc herniation)

**Emotion:** Two subthemes were identified: *emotions before and after physical activity* and *emotion-related obstacles.*

The subtheme *emotions before and after physical activity* described how emotional states fluctuated in relation to physical activity, influencing patients’ engagement. Before physical activity, many experienced emotional difficulties that acted as barriers, often rooted in the frustration of wanting to be active but being prevented by pain and the physical limitations described under skills and beliefs about capabilities. They described feeling *‘stuck in limbo’, ‘bummed’,* or that it had *‘screwed with my head’*. Importantly, these negative emotional states could also undermine patients’ confidence in their own abilities, reinforcing the reduced sense of capability described under beliefs about capabilities and creating a self-reinforcing cycle of inactivity. In contrast, after physical activity, patients reported positive emotional outcomes that acted as facilitators, saying they ‘*enjoy*’ it, felt a sense of *‘satisfaction’*, were *‘in a better mood’* or felt *‘a lot better’*.

“If I was overly sad or upset or feeling a little bit blue [or] depressed, I would not want to exercise.” (b) (Patient 1, 30–39 years, lumbar disc herniation)

“If you’re sad, and you go and do physical activities, you’re probably gonna end up with a smile on your face after an hour or so.” (f) (Patient 4, 70–79 years, lumbar stenosis)

The subtheme *emotion-related obstacles* reflected a deeper and more persistent emotional process than situation pre-activity feelings. This ongoing physical distress stemmed directly from patients’ chronic pain, functional limitations, and eroded physical ability, the same barriers that dominated beliefs about capabilities and skills, and acted as a sustained barrier to engagement. Many reported persistent low mood due to limitations in daily life, describing a ‘*depressive mindset’*, feeling ‘*depressing*’, and that it was ‘*very hard to accept that I couldn’t get out mentally to do things that I wanted to do’*. They also mentioned feeling ‘*frustrating’, ‘anxious’, ‘I’m missing out on my life’*, that it ‘*freaks me out’*, and *‘fear’* of pain and associated symptoms. Many missed being able to engage in physical activity without symptoms, highlighting how the intersection of physical and emotional burden created compounding barriers across multiple domains.

“Mentally, […] I think nobody wants to admit to it, but it is a big downer. Not being able to do what you want to do. That’s kind of […] being locked away in a prison, isn’t it?” (b) (Patient 4, 70–79 years, lumbar stenosis)

#### 2. Other domains.

Seven other relevant domains were identified: *knowledge; social/professional role and identity; motivation and goals; memory, attention and decision processes; social influences; behavioural regulation; and nature of the behaviours.*

**Knowledge:** One subtheme was identified: *limited or no guideline awareness*.

Patients reported *limited or no guideline awareness* about physical activity guidelines for adults, which acted as a barrier. Some cited partial but inaccurate knowledge, mentioning *‘4 to 10,000 steps a day’* or *‘30 minutes a day’*, without considering intensity, while others showed complete unawareness, responding ‘*not really’, ‘no idea’,* and *‘probably nothing’*.

“Recommendations? I couldn’t, I wouldn’t be able to put a number on it.” (b) (Patient 13, 50–59 years, lumbar disc herniation)

**Social/professional role and identity:** One subtheme was identified: *physical activity identity and past experiences.*

Patients with a *physical activity identity and past experiences* were physically active before found it easier to engage in physical activity now despite their back condition, acting as a facilitator. Some described physical activity as *‘very important’*, saw themselves as *‘physically active’*, an *‘outdoors guy’*, or viewed it as a *‘lifestyle’*. In contrast, those who considered physical activity a ‘*very little’* or *‘not much’* part of their lives, or no longer identified as active due to reduced levels after their condition, faced greater difficulties, acting as a barrier.

“I never had that in my younger 20s, or even when I was a teenager, it didn’t prepare me as an adult to be an active person or to know how to do things properly, [such as] to play certain sports. I never played sports when I was a teenager, like, all of these things actually do contribute to [being] an inactive adult.” (b) (Patient 1, 30–39 years, lumbar disc herniation)

“Always, […] I was a very active kid. Played a million sports right around with my cousins. Then, you know, [I] competed in university sports and then turned pro after [that], like, and then retired with expecting to just do different things. […]. And I like teaching because it’s also a very active profession.” (f) (Patient 8, 30–39 years, lumbar disc herniation)

**Motivation and goals:** One subtheme was identified: *motivation and personal goals despite difficulties.*

Many patients maintained *motivation and personal goals despite difficulties* and years of living with their condition, acting as a facilitator. Although motivation fluctuated, they tried to make the most of the days they felt capable. Notably, this motivation persisted despite the pain, associated symptoms, and functional limitations described under beliefs about capabilities and skills, making it a particularly salient facilitator in this population. Rather than abandoning physical activity altogether, patients said they *‘aspire’, ‘try’,* or were *‘looking forward’* to perform physical activity, aiming to be *‘as active as possible’* or return to their previous level.

“I have a hard time with motivation. I think that’s the biggest key cause. Yes, I want to do it and yes, I feel driven to do it, but it’s not all the time.” (f) (Patient 1, 30–39 years, lumbar disc herniation)

“Right now, I know the feeling that I can’t do it, and I want to do it. I don’t want to be sitting on my butt, I don’t want to be watching TV all the time. We’re stuck in front of a computer all day. I want to be out in the fresh air, biking, that type of thing, more walking.” (f) (Patient 4, 70–79 years, lumbar stenosis)

**Memory, attention and decision processes:** One subtheme was identified: *lack of deliberate regular reminders.*

Some patients reported a *lack of deliberate regular reminders*, which acted as a barrier. They relied on themselves to remember, saying ‘*it’s just me’, ‘don’t need to’,* or *‘when notion hits me*’, and often considered reminders unecessary because symptoms already prevented physical activity.

“At this point in time, I don’t remind myself as I can’t do it.” (b) (Patient 4, 70–79 years, lumbar stenosis)

**Social influences:** Two subthemes were identified: *family and friends* and *healthcare providers*.

*Family and friends* played an important role in patients’ physical activity, mainly as facilitators, motivating and often accompanying patients in activities like walking or going to the gym. Patients said their partners, children, or neighbors were *‘very supportive’* and *‘encourages me’*. However, overprotective attitudes, like being ‘*very protective*’, saying *‘doesn’t let me do much’*, or doing *‘lion’s share of the work’*, acted as barriers by limiting patients’ physical activity.

“As I said, my wife is […] like a sergeant, sergeant major. She tries to do everything for me.” (b) (Patient 14, 70–79 lumbar stenosis)

“Very well, like, everybody encourages me to get out there and do what I can and move. Yeah, I think that they are all very supportive of that.” (f) (Patient 1, 30–39 years, lumbar disc herniation)

*Healthcare providers* often acted as facilitators, especially family doctors, nurses, and physiotherapists, offering ‘*very helpful’* rehabilitation, *‘tips how to stretch’*, advice to *‘stay active’* or *‘be in shape’*, and home exercise programs. In contrast, a lack of information acted as a barrier, with patients saying they *‘can’t remember receiving anything’,* that *‘there’s not been a lot of information’*, or *healthcare providers ‘didn’t give me anything’*. Some received unclear information like *‘as active as tolerated’*, and inconsistent guidance across professionals was also mentioned as a barrier.

“You know, I talked to my chiropractor and they say, ‘well’. I talked to my doctor, they say another thing. My doctor recommends this book, and it’s about, like, fixing your own back or whatever, so I bought it, no problem. But I said to my chiropractor, ‘Do you believe in this book?’ He said ‘no’, I was so upset, I’m just like, ‘can nobody get on the same page?’.” (b) (Patient 12, 30–39 years, lumbar disc herniation)

“Every person I’ve run into, nurses, doctors, the staff here. Everybody’s extremely helpful, and a lot of people are overwork.” (f) (Patient 4, 70–79 years, lumbar stenosis)

**Behavioural regulation:** Three subthemes was identified: *alternative strategies and problem solving, monitoring and feedback,* and *action planning.*

Given the chronic nature of their condition, patients often used a*lternative strategies and problem solving* as facilitators to engage in physical activity. Grounded in personal experience rather than formal guidelines (consistent with the limited guideline awareness described under knowledge) patients adapted the intensity or type of activity when symptoms appeared, saying ‘*stop and then I’ll try again’* or *‘if my leg is bothering me tremendously, I won’t do the hills, we just walk’*. Some used medication to alleviate symptoms, and when weather was a barrier, they adjusted accordingly, for example, *‘if it’s raining, I put on a warmer coat and go out in the rain’* or *‘rearrange my schedule so that I do it at night’*.

“We do that pretty much every day unless it’s pouring rain, [and] then we’ll go to the mall and walk around in the mall.” (f) (Patient 6, 60–69 years, lumbar stenosis

*Monitoring and feedback* served as facilitators by helping patients understand their activity levels, set goals, and aim to ‘*go more than the last time, no matter what I did’*. Many people used smartwatches connected to smartphones. However, for some, tracking was also a barrier, as it *‘sounds too much like work’*.

“So, I’m not great at doing it at the time, but usually, like, at the end of the day, I can say what I did in the app, and it kind of gives me an idea, because it does have, like, little highlights of the days that you exercised, and the things that you did really well and hit your goals, etcetera.” (f) (Patient 1, 30–39 years, lumbar disc herniation)

*Action planning* was a facilitator, helping patients engage in physical activity by setting specific activities, schedules, intensities, and companions. Planning was closely informed by symptom patterns, with patients identifying optimal moments based on pain levels was lower or energy, saying ‘*the mornings are the best’* or *‘this morning I’m in really good shape, I’m gonna do this, and this, and this*’. This symptom-driven scheduling reflects the same experiential self-knowledge described under alternative strategies and problem solving, and anticipates the role of daily routines described under nature of the behaviours. For others, *action planning* was a barrier, as they considered it *‘not necessarily’* important.

“It’s not like I avoid it, but I certainly don’t plan on it.” (b) (Patient 12, 30–39 years, lumbar disc herniation)

“I have to plan day upon day that I don’t have too much scheduled in my day because I don’t tolerate the activity well. So, I have to make sure that I break it up over days in the week, yeah.” (f) (Patient 7, 60–69 years, lumbar stenosis)

**Nature of the behaviours:** One subtheme was identified: *daily routines and habits*.

*Daily routines and habits* were a facilitator for patients who regularly included physical activity in their daily lives, feeling ‘*easygoing’* and automatic, with no need to think about what, how, or when to do it. Many reported engaging in it *‘four times a week’, ‘every morning’, or ‘daily’.* These routines were often shaped by symptom patterns, with patients gravitating towards moments of lower pain, a process closely linked to the symptom-driven action planning described under behavioural regulation. However, *daily routines and habits* were also a barrier for those who had never developed them or who struggled to re-establish previous habits, reflecting how pain and functional limitations, as described under beliefs about capabilities, can disrupt even well-established behavioural patterns. Some said *‘this back injury happened’* and they *‘haven’t been able to do that since my back’s gotten worse’.*

“I was really, really active prior to that. I did [write] that down, I did gardening, walking, swimming, aquasize, curling, cross-country skiing, downhill skiing, snowshoeing and I love travel, yeah. And I was doing all that before it hurt my back. And since I hurt my back, it’s been […] pretty much erased.” (b) (Patient 7, 60–69 years, lumbar stenosis)

“We tend to go for a walk every evening, like, now it’s a pattern, it’s a habit.” (f) (Patient 12, 30–39 years, lumbar disc herniation)

## Discussion

### Principal findings

This study aimed to identify the barriers and facilitators influencing engagement in physical activity among patients with degenerative lumbar conditions awaiting surgery. These patients typically experienced moderate to severe pain and substantial disability, shaping their daily functioning and perceptions of physical activity. Overall, patients described their experiences as a continuous negotiation between limitations and facilitators, reflecting the complex and dynamic interplay of personal, emotional, and contextual factors that characterise physical activity behaviour in this population.

Physical restrictions appeared to be a dominant barrier. Back and leg pain, together with leg tingling, burning, or numbness, often shaped daily life and influenced activity levels [37]. Patients typically engaged in physical activity when symptoms were less intense, yet pain remained unpredictable and often overwhelming. No meaningful differences in perceived barriers or facilitators emerged between patients with lumbar stenosis and those with lumbar disc herniation, suggesting that the behavioural mechanisms identified here may apply broadly across degenerative lumbar conditions. Fear of symptom exacerbation frequently led to avoidance or modifications [38], creating a cycle where symptoms restricted participation and inactivity further contributed to deconditioning. This pattern reflects a self-reinforcing experience where multiple TDF domains coexist. Specifically, pain and functional limitation co-occurred with diminished beliefs about capabilities, negative beliefs about consequences, an erosion of the physical skills needed to remain physically active, and persistent emotional distress. Impaired balance, reduced endurance, and comorbidities further limited engagement. At the same time, patients expressed clear benefits of physical activity, such as better mood, sleep, weight control, and strength [39], yet these were often overshadowed by the fear of aggravation, suggesting that interventions need to address not only physical capacity but also patients’ beliefs about the safety and consequence of physical activity. Guidance from health professionals played a pivotal role: lack of instruction generated uncertainty and fear, whereas appropriate guidance increased confidence and safety.

Contextual circumstances also shaped engagement. Access to safe spaces and natural environments facilitated activity, while financial limitations, inadequate facilities, and reduced healthcare access acted as barriers. Household and work responsibilities had a dual role, serving as the main source of activity for some but leaving others with little time or energy for it. Weather conditions were similarly ambivalent, as poor weather discouraged outdoor activity and, for patients already managing balance difficulties, heightened the perceived risk of falls and injury.

Emotional burden was closely tied to symptoms. Living for years with the condition was often accompanied by reports of persistent sadness, frustration, and fear, which discouraged participation [39]. Nevertheless, many patients described a marked improvement in mood, satisfaction, and well-being following physical activity, highlighting the tension between emotional barriers that preceded physical activity and the benefits experienced once it was completed.

Given the chronic nature of the condition, patients developed personalised, experience-based strategies to cope with symptom fluctuations and remain active within their physical limits [40], largely unaware of formal physical activity guidelines. Planning and monitoring activities further promoted adherence and fostered motivation by helping patients track their progress and strive for improvement over time. Patients tended to schedule activity at moments when symptoms were lower, integrating symptom management into their daily routines in a way that reflects the close relationship between behavioural regulation and habit formation.

Social influences generally acted as facilitators. Encouragement from family, friends, and healthcare providers supported engagement, although overprotection from relatives and conflicting advice from health professionals sometimes created barriers. Motivation and personal goals facilitated many patients to engage in physical activity despite their limitations [41], and this persistence is itself a clinically relevant finding, suggesting that the desire to remain active often endures even when capability is reduced. Daily routines and habits were seen as facilitating the integration of physical activity into everyday life, whereas disruptions to routines made re-establishing activity seem challenging, underscoring the importance of continuity and habit formation in this population.

### Strengths and limitations

This study had several strengths. It provided a patient-centered perspective on engagement in physical activity in individuals with degenerative lumbar spinal conditions awaiting surgery, including patients regardless of personal or contextual circumstances. Semi-structured interviews enabled a comprehensive exploration of barriers and facilitators [30], and the use of the TDF ensured a structured and theory-driven analysis [19]. The inclusion of both lumbar stenosis and lumbar disc herniation captured a broader and clinically meaningful range of experiences, enriching the findings. A patient representative contributed to the development of the topic guide and assisted with the interpretation of the findings, and the topic guide was piloted twice before recruitment.

Some limitations should be acknowledged. First, the study was conducted at a single center, which, combined with the lack of racial and ethnic diversity, may restrict the transferability of the results to different healthcare systems and more diverse populations. The recruitment process may also have been subject to self-selection bias, as patients more interested in physical activity might have been more likely to volunteer. The inclusion of two different diagnoses could have introduced variability in experiences, and the imbalance between lumbar stenosis and lumbar disc herniation cases may have influenced the relative weight of perspectives. Reliance on self-reported data also entails a risk of recall or response bias [42], and the absence of objective measures of physical activity, such as accelerometers, means that reported activity levels could not be verified. The study was based solely on patient interviews, without triangulation from other sources such as healthcare providers or observational data, which limits the depth and corroboration of the findings. No systematic data were collected on non-participating patients, precluding a formal comparison between participants and non-participants and limiting our ability to assess the representativeness of the sample. Finally, formal inter-rater reliability metrics were not calculated. Although coding decisions were guided by the prespecified categories of the TDF framework, the absence of a formal index is acknowledged as a limitation.

### Relation to other studies

Our findings are consistent with previous qualitative research in patients with lumbar stenosis and lumbar disc herniation. As in earlier work [17], symptoms, particularly pain, emerged as a key barrier, shaping both participation in structured rehabilitation and daily activities. The self-reinforcing cycle identified here, whereby pain drives inactivity, which in turn worsens deconditioning and comorbidities, extends previous observations by illustrating the mechanisms through which this cycle operates across multiple behavioural domains simultaneously. Mixed perceptions of physical activity were also consistent with prior studies: while some patients experienced physical and mental benefits [17,43], others considered it of limited personal value, especially when presented as an alternative to surgery [43]. By broadening the focus from prehabilitation to physical activity in general, our study extends these observations and shows how barriers and facilitators operate not only in hospital-based programs but also in everyday life.

Professional guidance emerged as a key facilitator across studies. One study highlighted the importance of structured rehabilitation [17], whereas our patients and another study in a rehabilitation setting [43] emphasised the need for consistent and coordinated information across different professionals and contexts. The finding that patients relied primarily on personal experiential knowledge rather than formal guidelines adds a new dimension, suggesting that improving guideline awareness and professional communication may be particularly impactful in this population.

Environmental and motivational factors were similarly with prior work [17]. Lack of time, financial limitations, and restricted access to safe environments hindered physical activity, and our study further highlighted the ambivalent role of household and work responsibilities. Motivation fluctuated over time, and our findings add that its persistence despite physical barriers is a clinically meaningful finding that interventions should seek to harness.

Findings from broader reviews in musculoskeletal [44,45] and chronic low back pain [46] populations were also largely consistent with ours. Pain aggravated by comorbidities was a prominent barrier, and both fear of injury and perceived benefits were reported across reviews. Relatives and healthcare professional emerged as important facilitators, and the role of positive emotions and habit formation was similarly underscored.

### Implications and future research

Beyond identifying individual barriers and facilitators, the findings suggest the presence of distinct conceptual profiles of engagement in physical activity, based on patterns observed across interviews. Three broad groups can be described: patients with low physical fitness and high perceived barriers who are unlikely to change their behaviour prior to surgery; patients with higher fitness and strong facilitators who can maintain activity despite symptoms; and patients with low fitness but openness to change if barriers and facilitators are adequately addressed. The identification of these profiles was not a formal aim of the study. These profiles are not intended as fixed categories but as a heuristic to illustrate population heterogeneity and inform the starting point for individualised clinical assessment, recognising that any intervention must ultimately be tailored to the individual, as discussed below. Future research should explore whether these profiles can be validated and whether they predict outcomes or response to intervention.

Our study suggests that personal, emotional, and contextual factors must be considered when promoting physical activity in patients awaiting surgery for degenerative lumbar spinal conditions. Given the multifactorial nature of the barriers and facilitators identified, prehabilitation interventions should be tailored to each patient’s perceived barriers and facilitators, their beliefs about physical activity and its consequences, and their emotional relationship with it. This initial conversation would also benefit from addressing symptom-specific concerns, distinguishing between lumbar and leg symptoms, and to explore patients’ own views on what kind of support would be most helpful. Such an approach aligns with the TDF domains identified as key in this study, and would allow clinicians to tailor recommendations to each patient’s specific profile. Specific behaviour change techniques and general approaches could then be mapped onto each patient’s profile. Beliefs about capabilities and consequences may be addressed through motivational interviewing, which incorporates strategies such as reframing physical activity as safe and beneficial [47]. Emotional barriers suggest the value of acceptance-based strategies to manage fear and frustration [48]. Skills-based limitations support the use of supervised, graded physical activity programs that rebuild confidence through progressive practice [49].

More broadly, intervention should aim to address the self-reinforcing cycle of pain, inactivity, and deconditioning by supporting patients in identifying safe and tolerable activity levels and providing clear, consistent guidance from healthcare professional [50]. Given the prominence of environmental barriers, home-based or hybrid delivery formats should be considered alongside strengthening individual facilitators such as social support [51]. Building and maintaining daily routines and habits appears particularly important, given how difficult patients found it to re-establish activity once disrupted. Structured action planning could support habit formation during the preoperative period, while self-monitoring tools such as activity trackers may help patients during the initial stages before behaviours become automatised [50].

These findings can inform surgeons, family doctors, physiotherapists, and relatives in supporting patients, and may guide healthcare managers in prioritizing strategies to promote physical activity in this population. Future research should include qualitative studies exploring the perspectives of surgeons, family doctors, and physiotherapists on preoperative physical activity. Feasibility studies are needed to test interventions that address the barriers and facilitators identified here, ideally including a control group and objective measures of physical activity such as accelerometers. Given that this population is typically older, future studies should test age-adapted programs that account for comorbidities and functional limitations while promoting safety and adherence. Interventions should also aim to strengthen outcomes highly relevant in older adults, such as independence in daily activities, fall prevention, reduction of frailty, and enhanced social engagement. Future research with larger, diagnosis-specific samples should also examine whether the barriers and facilitators identified here differ between lumbar stenosis and lumbar disc herniation, given that the present study was not powered to detect such differences.

## Conclusion

Engagement in physical activity among patients with degenerative spinal conditions awaiting spine surgery is shaped by an interplay of physical, emotional, and contextual factors. By applying the TDF, this study provides a theoretically grounded understanding of these barriers and facilitators. This pattern reveals a self-reinforcing experience where multiple TDF domains coexist: pain and functional limitation co-occurr with diminished beliefs about capabilities, negative beliefs about consequences, an erosion of the physical skills needed to remain physically active, and persistent emotional distress. Rather than applying generic activity recommendations, clinicians should begin with an individualised assessment to explore each patient’s barriers, facilitators, beliefs, and symptom-specific concerns, and tailor support accordingly, with particular attention to professional guidance, habit formation, and emotional support. Future research should test the feasibility of theory-driven interventions informed by these findings and explore the perspectives of healthcare providers to ensure that strategies are both effective and applicable in practice.

## Supporting information

S1 File Topic guide. (PDF)

S2 File Physical activity prompt. (PDF)

S3 File COREQ checklist. (PDF)

S4 File Criteria for identifying TDF domains as. key. (PDF)

S1 Table Number of quotes for each domain. (PDF)

## Abbreviations

BMI: Body Mass Index
CSORN: Canadian Spine Outcomes and Research Network
COREQ: Consolidated Criteria for Reporting Qualitative Research
EQ-VAS: EuroQol Visual Analogue Scale
GSLTPAQ: Godin-Shephard Leisure-Time Physical Activity Questionnaire
mODI: modified Oswestry Disability Index
SD: standard deviation
TDF: Theoretical Domains Framework

## References

[1] Ravindra VM, Senglaub SS, Rattani A, et al. Degenerative lumbar spine disease: estimating global incidence and worldwide volume. Global Spine J. 2018;8(8):784–94. doi: 10.1177/2192568218770769 30560029 PMC6293435

[2] Thivel D, Tremblay A, Genin PM, Panahi S, Rivière D, Duclos M. Physical activity, inactivity, and sedentary behaviors: definitions and implications in occupational health. Front Public Health. 2018;6:288. doi: 10.3389/fpubh.2018.00288 30345266 PMC6182813

[3] GBD 2021 Low Back Pain Collaborators. Global, regional, and national burden of low back pain, 1990–2020, its attributable risk factors, and projections to 2050: a systematic analysis of the Global Burden of Disease Study 2021. Lancet Rheumatol. 2023;5(6):e316–29. doi: 10.1016/S2665-9913(23)00098-X PMC10234592 37273833

[4] Ishimoto Y, Yoshimura N, Muraki S, Yamada H, Nagata K, Hashizume H, et al. Associations between radiographic lumbar spinal stenosis and clinical symptoms in the general population: the Wakayama Spine Study. Osteoarthr Cartil. 2013;21(6):783–8. doi: 10.1016/j.joca.2013.02.656 23473979

[5] Otani K, Kikuchi S, Yabuki S, Igarashi T, Nikaido T, Watanabe K, et al. Lumbar spinal stenosis has a negative impact on quality of life compared with other comorbidities: an epidemiological cross-sectional study of 1862 community-dwelling individuals. Sci World J. 2013;2013:590652. doi: 10.1155/2013/590652 24453878 PMC3885225

[6] Pearson A. Spinal stenosis - symptoms, diagnosis and treatment | BMJ Best Practice US. Available from: https://bestpractice.bmj.com/topics/en-us/191

[7] Awadalla AM, Aljulayfi AS, Alrowaili AR, Souror H, Alowid F, Mahdi AMM, et al. Management of lumbar disc herniation: a systematic review. Cureus. 2023;15(10):e47908. doi: 10.7759/cureus.47908 38034203 PMC10683841

[8] Hasvik E, Haugen AJ, Grøvle L. Symptom descriptors and patterns in lumbar radicular pain caused by disc herniation: a 1-year longitudinal cohort study. BMJ Open. 2022;12(12):e065500. doi: 10.1136/bmjopen-2022-065500 36549718 PMC9772640

[9] Wang J, Zou Q, Li S, Tang R, Yang X, Zeng J, et al. Gait asymmetry of lower extremities reduced immediately after minimally invasive surgery among patients with lumbar disc herniation. Clin Biomech (Bristol). 2022;98:105720. doi: 10.1016/j.clinbiomech.2022.105720 35863143

[10] Hebert JJ, Abraham E, Wedderkopp N, Bigney E, Richardson E, Darling M, et al. Patients undergoing surgery for lumbar spinal stenosis experience unique courses of pain and disability: a group-based trajectory analysis. PLoS One. 2019;14(11):e0224200. doi: 10.1371/journal.pone.0224200 31697714 PMC6837529

[11] Wang S, Hebert JJ, Abraham E, Vandewint A, Bigney E, Richardson E, et al. Postoperative recovery patterns following discectomy surgery in patients with lumbar radiculopathy. Sci Rep. 2022;12(1):11146. doi: 10.1038/s41598-022-15169-8 35778472 PMC9249755

[12] Doth AH, Hansson PT, Jensen MP, Taylor RS. The burden of neuropathic pain: a systematic review and meta-analysis of health utilities. Pain. 2010;149(2):338–44. doi: 10.1016/j.pain.2010.02.034 20227832

[13] Taylor RS, Taylor RJ. The economic impact of failed back surgery syndrome. Br J Pain. 2012;6(4):174–81. doi: 10.1177/2049463712470887 26516490 PMC4590097

[14] Global health risks: mortality and burden of disease attributable to selected major risks. 2009:16–8.

[15] Hébert JJ, Abraham E, Wedderkopp N, Bigney E, Richardson E, Darling M, et al. Preoperative factors predict postoperative trajectories of pain and disability following surgery for degenerative lumbar spinal stenosis. Spine (Phila Pa 1976). 2020;45(21):E1421–30. doi: 10.1097/BRS.0000000000003587 32541610 PMC7547903

[16] Tschugg A, Lener S, Hartmann S, Wildauer M, Löscher WN, Neururer S, et al. Preoperative sport improves the outcome of lumbar disc surgery: a prospective monocentric cohort study. Neurosurg Rev. 2017;40(4):597–604. doi: 10.1007/s10143-017-0811-6 28091825 PMC5591354

[17] Lindbäck Y, Carlfjord S. Experiences from pre-surgery physiotherapy and thoughts about future exercise among patients with disc herniation or spinal stenosis: a qualitative study. Musculoskelet Sci Pract. 2024;69:102892. doi: 10.1016/j.msksp.2023.102892 38070465

[18] Lotzke H, Jakobsson M, Gutke A, Hagströmer M, Brisby H, Hägg O, et al. Patients with severe low back pain exhibit a low level of physical activity before lumbar fusion surgery: a cross-sectional study. BMC Musculoskelet Disord. 2018;19(1):365. doi: 10.1186/s12891-018-2274-5 30305065 PMC6180521

[19] Michie S, Johnston M, Abraham C, Lawton R, Parker D, Walker A, et al. Making psychological theory useful for implementing evidence based practice: a consensus approach. Qual Saf Health Care. 2005;14(1):26–33. doi: 10.1136/qshc.2004.011155 15692000 PMC1743963

[20] Atkins L, Francis J, Islam R, O’Connor D, Patey A, Ivers N, et al. A guide to using the Theoretical Domains Framework of behaviour change to investigate implementation problems. Implement Sci. 2017;12(1):77. doi: 10.1186/s13012-017-0605-9 28637486 PMC5480145

[21] Michie S, van Stralen MM, West R. The behaviour change wheel: a new method for characterising and designing behaviour change interventions. Implement Sci. 2011;6:42. doi: 10.1186/1748-5908-6-42 21513547 PMC3096582

[22] King LK, Krystia O, Waugh EJ, MacKay C, Stanaitis I, Stretton J, et al. Barriers and enablers to health care providers assessment and treatment of knee osteoarthritis in persons with type 2 diabetes mellitus: a qualitative study using the Theoretical Domains Framework. Osteoarthr Cartil Open. 2022;4(4):100299. doi: 10.1016/j.ocarto.2022.100299 36474789 PMC9718241

[23] Gray EA, Skinner MA, Hale LA. Perceived facilitators of physical activity following coronary artery bypass graft surgery: a qualitative study. Cardiopulm Phys Ther J. 2024;35(2):38–49. doi: 10.1097/cpt.0000000000000240

[24] Amirova A, Lucas R, Cowie MR, Haddad M. Perceived barriers and enablers influencing physical activity in heart failure: a qualitative one-to-one interview study. PLoS One. 2022;17(8):e0271743. doi: 10.1371/journal.pone.0271743 35925964 PMC9352074

[25] Ford S, Schofield T, Hope T. Are patients’ decision‐making preferences being met? Health Expect. 2003;6(1):72–80. doi: 10.1046/j.1369-6513.2003.00211.x 12603630 PMC5060161

[26] Renjith V, Yesodharan R, Noronha JA, Ladd E, George A. Qualitative methods in health care research. Int J Prev Med. 2021;12:20. doi: 10.4103/ijpvm.IJPVM_321_19 34084317 PMC8106287

[27] Yao M, Xu B-P, Li Z-J, Zhu S, Tian Z-R, Li D-H, et al. A comparison between the low back pain scales for patients with lumbar disc herniation: validity, reliability, and responsiveness. Health Qual Life Outcomes. 2020;18(1):175. doi: 10.1186/s12955-020-01403-2 32522196 PMC7288427

[28] Fritz JM, Irrgang JJ. A comparison of a modified Oswestry Low Back Pain Disability Questionnaire and the Quebec Back Pain Disability Scale. Phys Ther. 2001;81(2):776–88. doi: 10.1093/ptj/81.2.776 11175676

[29] EuroQol Group. EuroQol--a new facility for the measurement of health-related quality of life. Health Policy. 1990;16(3):199–208. doi: 10.1016/0168-8510(90)90421-9 10109801

[30] Kwasnicka D, Dombrowski SU, White M, Sniehotta FF. Data-prompted interviews: using individual ecological data to stimulate narratives and explore meanings. Health Psychol. 2015;34(12):1191–4. doi: 10.1037/hea0000234 26010718 PMC4671473

[31] Ross R, Chaput J-P, Giangregorio LM, Janssen I, Saunders TJ, Kho ME, et al. Canadian 24-Hour Movement Guidelines for Adults aged 18-64 years and Adults aged 65 years or older: an integration of physical activity, sedentary behaviour, and sleep. Appl Physiol Nutr Metab. 2020;45(10 (Suppl. 2)):S57–102. doi: 10.1139/apnm-2020-0467 33054332

[32] Godin G. The Godin-Shephard leisure-time physical activity questionnaire. HFJC. 2011;4(1):1. doi: 10.14288/hfjc.v4i1.82

[33] Hennink M, Kaiser BN. Sample sizes for saturation in qualitative research: a systematic review of empirical tests. Soc Sci Med. 2022;292:114523. doi: 10.1016/j.socscimed.2021.114523 34785096

[34] Francis JJ, Johnston M, Robertson C, Glidewell L, Entwistle V, Eccles MP, et al. What is an adequate sample size? Operationalising data saturation for theory-based interview studies. Psychol Health. 2010;25(10):1229–45. doi: 10.1080/08870440903194015 20204937

[35] Tong A, Sainsbury P, Craig J. Consolidated criteria for reporting qualitative research (COREQ): a 32-item checklist for interviews and focus groups. Int J Qual Health Care. 2007;19(6):349–57. doi: 10.1093/intqhc/mzm042 17872937

[36] Cofie N, Braund H, Dalgarno N. Eight ways to get a grip on intercoder reliability using qualitative-based measures. Can Med Educ J. 2022;13(2):73–6. doi: 10.36834/cmej.72504 35572014 PMC9099179

[37] Ryan CG, Grant PMM, Dall PM, Gray H, Newton M, Granat MH. Individuals with chronic low back pain have a lower level, and an altered pattern, of physical activity compared with matched controls: an observational study. Aust J Physiother. 2009;55(1):53–8. doi: 10.1016/s0004-9514(09)70061-3 19226242

[38] Yihunie M, Abich Y, Demissie SF, Kassa T, Ranganathan P, Janakiraman B. Fear-avoidance beliefs for physical activity among chronic low back pain: a multicenter cross-sectional study. J Pain Res. 2023;16:233–43. doi: 10.2147/JPR.S388002 36726857 PMC9885961

[39] Karlsson L, Gerdle B, Takala E-P, Andersson G, Larsson B. Experiences and attitudes about physical activity and exercise in patients with chronic pain: a qualitative interview study. J Pain Res. 2018;11:133–44. doi: 10.2147/JPR.S149826 29379314 PMC5759850

[40] Bourke MJ, Ferguson D, Cooke M. Patient experiences of self-management for chronic low back pain: a qualitative study. Phys Ther. 2022;102(6):pzac030. doi: 10.1093/ptj/pzac030 35358311

[41] Nevelikova M, Zlamal F, Dosbaba F, Su JJ, Batalik L. Motivation to exercise in patients with chronic low back pain. BMC Musculoskelet Disord. 2025;26(1):226. doi: 10.1186/s12891-025-08461-x 40050856 PMC11883927

[42] Althubaiti A. Information bias in health research: definition, pitfalls, and adjustment methods. J Multidiscip Healthc. 2016;9:211–7. doi: 10.2147/JMDH.S104807 27217764 PMC4862344

[43] McCarthy AE, Bove AM, Piva S, Mecca LP, Schneider MJ. A qualitative study of preparation for lumbar spinal stenosis surgery: perceptions of patients and physical therapists. J Orthop Sports Phys Ther. 2020;50(4):198–205. doi: 10.2519/jospt.2020.8887 31663813

[44] Booth G, D’Lima D, Gilbert A, Greenwood J, Sharma N, Howarth A, et al. Barriers and facilitators to physical activity for people with persistent musculoskeletal pain: systematic review and synthesis using the Theoretical Domains Framework. Eur J Physiother. 2023;26(5):299–312. doi: 10.1080/21679169.2023.2276714

[45] Webb J, Baker A, Palmer T, Hall A, Ahlquist A, Darlow J, et al. The barriers and facilitators to physical activity in people with a musculoskeletal condition: a rapid review of reviews using the COM-B model to support intervention development. Public Health Pract (Oxf). 2022;3:100250. doi: 10.1016/j.puhip.2022.100250 36101772 PMC9461378

[46] Grande GHD, Vidal RVC, Salini MCR, Christofaro DGD, Oliveira CB. Barriers and facilitators to physical activity and exercise among people with chronic low back pain: a qualitative evidence synthesis. J Orthop Sports Phys Ther. 2025;55(5):312–30. doi: 10.2519/jospt.2025.12905 40298245

[47] Gilbert AL, Lee J, Ehrlich-Jones L, Semanik PA, Song J, Pellegrini CA, et al. A randomized trial of a motivational interviewing intervention to increase lifestyle physical activity and improve self-reported function in adults with arthritis. Semin Arthritis Rheum. 2018;47(5):732–40. doi: 10.1016/j.semarthrit.2017.10.003 29096934 PMC5866185

[48] Veehof MM, Oskam M-J, Schreurs KMG, Bohlmeijer ET. Acceptance-based interventions for the treatment of chronic pain: a systematic review and meta-analysis. Pain. 2011;152(3):533–42. doi: 10.1016/j.pain.2010.11.002 21251756

[49] Marchand A-A, Houle M, O’Shaughnessy J, Châtillon C-É, Cantin V, Descarreaux M. Effectiveness of an exercise-based prehabilitation program for patients awaiting surgery for lumbar spinal stenosis: a randomized clinical trial. Sci Rep. 2021;11(1):11080. doi: 10.1038/s41598-021-90537-4 34040109 PMC8155114

[50] Vaegter HB, Kinnunen M, Verbrugghe J, Cunningham C, Meeus M, Armijo-Olivo S, et al. Physical activity should be the primary intervention for individuals living with chronic pain A position paper from the European Pain Federation (EFIC) “On the Move” Task Force. Eur J Pain. 2024;28(8):1249–56. doi: 10.1002/ejp.2278 38703009

[51] Palazzo C, Klinger E, Dorner V, Kadri A, Thierry O, Boumenir Y, et al. Barriers to home-based exercise program adherence with chronic low back pain: patient expectations regarding new technologies. Ann Phys Rehabil Med. 2016;59(2):107–13. doi: 10.1016/j.rehab.2016.01.009 27050664

