# Supplementary material for: Physical activity barriers and facilitators in patients awaiting spine surgery: A qualitative study
Source: PLoS One. 2026 Aug 4;21(8):e0355173. doi: 10.1371/journal.pone.0355173 (PMC13436774; doi:10.1371/journal.pone.0355173)
Supplement: Supplementary file 1 [file pone.0355173.s001.pdf]

Barriers & Enablers Influencing Physical Activity
TDF Interview

Patient ID: Interview Date:

Domains	Questions
1. Knowledge	1. What physical activity do you do at the moment?<br>*1.1. Prompt: On a typical day, what physical activity do you do? Can you give other examples of physical activity that you do?*<br>2. What do you know about the recommendations for physical activity for adults?<br>*2.1. Prompt of recommended time: Canada recommends 150 minutes of moderate to vigorous intensity physical activity per week.*<br>*2.2. Prompt of recommended intensity: Examples. (Adapt examples to PA ability)*<br>*2.3. Prompt: Physical activity is also recommended for patients who are on the waiting list for spinal surgery.*<br>3. What are your thoughts about these recommendations?

	Hand shaking		Difficulty to communicate		Emotional response		Restelssness or agitation		Sadness or hopeless

Other:
*Prompt: For the following questions, we will now consider your pursuits that contribute to the time you spend doing **moderate to vigorous intensity** activities.*

2. Skills	1. How easy or difficult do you find doing physical activity?<br>2. What specific skills do you need to engage in physical activity?<br>*2.1. Prompt: balance, strength, etc.*<br>3. Have your skills to do physical activity changed over time?<br>*3.1. Prompt: Improved or worsened? What skills?*

	Hand shaking		Difficulty to communicate		Emotional response		Restelssness or agitation		Sadness or hopeless

Other:

Version 2.0, 30-Jul-2024

3. Social /professional role and identity	1. How much is physical activity part of your daily life?<br>2. Do you see yourself as a physically active person?<br>2.1. Prompt: if not. Could you see yourself becoming a physically active person?								
	Hand shaking		Difficulty to communicate		Emotional response		Restlessness or agitation		Sadness or hopeless
Other:									
4. Beliefs about capabilities (self-efficacy)	1. Do you think that doing physical activity is within your control?<br>*1.1. Prompt: Do you feel you can be physically active on your own or do you depend on someone or something to be active?*<br>2. How confident are you that you could be physically active if you wanted to?								
	Hand shaking		Difficulty to communicate		Emotional response		Restlessness or agitation		Sadness or hopeless
Other:									
5. Beliefs about consequences	1. In your mind, what are the results of doing physical activity?<br>*1.1 Benefits: feel better, sleep better, heal the spine, become more independent*<br>*1.2 Consequences: be more tired the rest of the day, accidental fall, injure yourself*<br>*2.* What are the results of not doing physical activity?<br>*1.1 Benefits: I will protect my spine, my spine will heal faster*<br>*1.2 Consequences: your spine could get worse, you might injure yourself more easily, your back condition may worsen*								
	Hand shaking		Difficulty to communicate		Emotional response		Restlessness or agitation		Sadness or hopeless
Other:									

6. Motivation and goals	1. How motivated are you to do physical activity?<br>2. Considering all the things you do in your daily life, how important is doing physical activity to you?

	Hand shaking		Difficulty to communicate		Emotional response		Restlessness or agitation		Sadness or hopeless

Other: a bit of emotional response remembering last year (rained so much)

7. Memory, attention, and decision processes	1. How do you decide when to do physical activity and what to do?<br>*1.1. Prompt: Think about what usually makes you decide to do physical activity. Is it the time you have free, how you are feeling that day, or what material you have available? What things matter most when you choose what to do and when?*<br>*2.* How do you remind yourself to do physical activity*?*<br>*1.1. Prompts: the use of calendars, mobile reminders, smartwatch reminders, etc.*

	Hand shaking		Difficulty to communicate		Emotional response		Restlessness or agitation		Sadness or hopeless

Other:

8. Environmental context and resources (environmental constraints)	1. What aspects of your surroundings help you to do physical activity?<br>*1.1. Prompts: Think about elements like nice weather, easy places to walk, or having company for physical activity. How do these factors help you to be physically active?*<br>2. What aspects of your surroundings make it more difficult to do physical activity?<br>*2.2. Prompts: Consider challenges such as bad weather, lack of places to walk, or absence of company for physical activity. Do any of these factors make it difficult for you to be physically active?*<br>3. To what extent do financial considerations impact you doing physical activity?

	Hand shaking		Difficulty to communicate		Emotional response		Restlessness or agitation		Sadness or hopeless

<table>
<tr><td colspan="10">Other:</td></tr>
<tr><td colspan="2">9. Social influences (Norms)</td><td colspan="8">1. What information do you remember receiving from your healthcare providers about doing physical activity while you are waiting for surgery?
2. How supported do you feel by your family, friends, and healthcare providers about doing physical activity?
3. Do you feel other people expect you to do physical activity?
3.1. Prompt: who?
4. How much do you think other people waiting for surgery are doing physical activity?</td></tr>
<tr><td></td><td>Hand shaking</td><td></td><td>Difficulty to communicate</td><td></td><td>Emotional response</td><td></td><td>Restlessness or agitation</td><td></td><td>Sadness or hopeless</td></tr>
<tr><td colspan="10">Other:</td></tr>
<tr><td colspan="2">10. Emotion</td><td colspan="8">1. What feelings encourage you to do physical activity?
1.1. Prompts: happiness, energy, etc. Please share any specific feelings that make you feel like being active.
2. What feelings discourage you from doing physical activity?
2.1. Prompts: for example, fear, worry, dread, discomfort, etc.
3. What specific physical activities do you enjoy?</td></tr>
<tr><td></td><td>Hand shaking</td><td></td><td>Difficulty to communicate</td><td></td><td>Emotional response</td><td></td><td>Restlessness or agitation</td><td></td><td>Sadness or hopeless</td></tr>
<tr><td colspan="10">Other:</td></tr>
<tr><td colspan="2">11. Behavioural regulation</td><td colspan="8">1. Do you sometimes make specific plans to do physical activity?
2. How do you track your physical activity levels?
2.1. Prompt: using a smartwatch, a mobile app, etc.
3. How do you deal with barriers that might get in the way of doing physical activity?</td></tr>
</table>

	Hand shaking		Difficulty to communicate		Emotional response		Restlessness or agitation		Sadness or hopeless
Other: 11.3 Ear touching and bit emotional response (10.15h)									
12. Nature of the behaviours	1. Are there specific behaviours or daily routines related to doing physical activity that you find particularly challenging or satisfying?<br>2. We have talked a lot about physical activity but is there anything else that we haven't discussed that you want to say?								
	Hand shaking		Difficulty to communicate		Emotional response		Restlessness or agitation		Sadness or hopeless
Other:									
