# Supplementary material for: Physical activity barriers and facilitators in patients awaiting spine surgery: A qualitative study
Source: PLoS One. 2026 Aug 4;21(8):e0355173. doi: 10.1371/journal.pone.0355173 (PMC13436774; doi:10.1371/journal.pone.0355173)
Supplement: Supplementary file 2 [file pone.0355173.s002.pdf]

PHYSICAL ACTIVITY

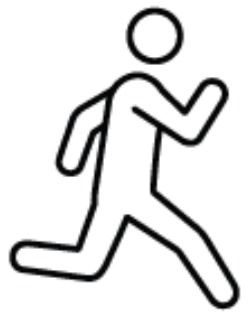

150 minutes per week
of moderate to vigorous
aerobic physical activities

22 minutes per day

LIGHT PHYSICAL ACTIVITY

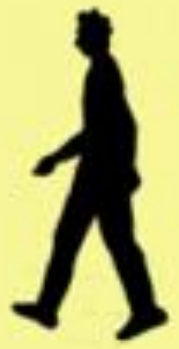

Slow walking

MODERATE PHYSICAL ACTIVITY

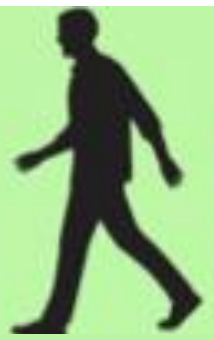

Brisk walking

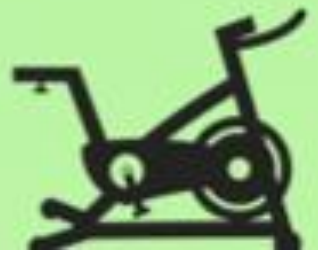

Cycling

VIGOROUS PHYSICAL ACTIVITY

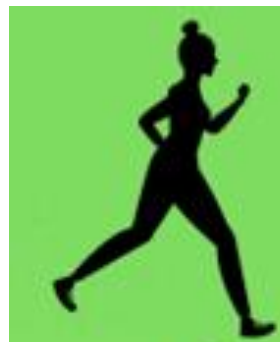

Jogging

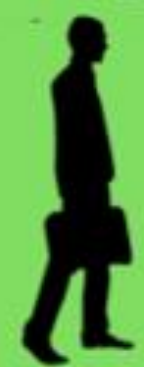

Walking with load

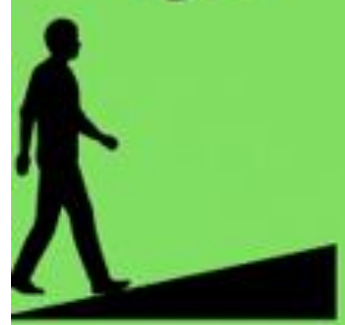

Walking uphill

Source:

- Light physical activity: Bull et al. 2020 World Health Organization 2020 guidelines on physical activity and sedentary behaviour.
- Moderate and vigorous physical activity: Canadian Physical Activity Guidelines Canadian Sedentary Behaviour Guidelines. Your Plan to Get Active Every Day and American heart association (https://www.heart.org/en/healthy-living/fitness/fitness-basics/aha-recs-for-physical-activity-in-adults)
