# Supplementary material for: Physical activity barriers and facilitators in patients awaiting spine surgery: A qualitative study
Source: PLoS One. 2026 Aug 4;21(8):e0355173. doi: 10.1371/journal.pone.0355173 (PMC13436774; doi:10.1371/journal.pone.0355173)
Supplement: Supplementary file 4 [file pone.0355173.s004.pdf]

Table 1. Criteria for identifying TDF domains as key to understanding the barriers of physical activity in patients with lumbar degenerative disease

TDF domain	(i) High frequency of specific beliefs indicating barrier	(ii) Indication of clear beliefs indicating barrier	(iii) Existence of conflicting beliefs
Knowledge	✓		
Skills	✓	✓	
Social/professional role and identity			✓
Beliefs about capabilities	✓	✓	
Beliefs about consequences	✓	✓	✓
Motivation and goals			
Memory, attention, and decision processes			
Environmental context and resources	✓	✓	✓
Social influences			✓
Emotion	✓	✓	✓
Behavioural regulation			
Nature of the behaviours			

Table 2. Criteria for identifying TDF domains that facilitate physical activity in patients with lumbar degenerative disease.

TDF domain	(i) High frequency of specific beliefs indicating facilitator	(ii) Indication of clear beliefs indicating facilitator	(iii) Existence of conflicting beliefs
Knowledge			
Skills			
Social/professional role and identity			✓
Beliefs about capabilities			
Beliefs about consequences	✓		✓
Motivation and goals	✓	✓	
Memory, attention, and decision processes			
Environmental context and resources	✓	✓	✓
Social influences	✓	✓	✓
Emotion	✓	✓	✓
Behavioural regulation	✓	✓	
Nature of the behaviours	✓	✓	
