# Supplementary material for: Physical activity barriers and facilitators in patients awaiting spine surgery: A qualitative study
Source: PLoS One. 2026 Aug 4;21(8):e0355173. doi: 10.1371/journal.pone.0355173 (PMC13436774; doi:10.1371/journal.pone.0355173)
Supplement: Supplementary file 5 [file pone.0355173.s005.pdf]

S1 Table. Number of quotes for each domain

TDF Domain	Facilitators (n quotes)	Barriers (n quotes)	Total (n quotes)
1. Knowledge	1	17	18
2. Skills	10	41	51
3. Social/professional role and identity	37	17	54
4. Beliefs about capabilities	21	146	167
5. Beliefs about consequences	56	78	134
6. Motivation and goals	53	5	58
7. Memory, attention, and decision processes	12	13	25
8. Environmental context and resources	89	53	142
9. Social influences	101	25	126
10. Emotion	47	60	107
11. Behavioural regulation	69	14	83
12. Nature of the behaviours	34	11	45

Note: The total number of quotes reflects all coded statements across participants. Multiple quotes from the same participant may be coded within the same domain, as a single patient could express several distinct beliefs relevant to that domain.
