# Supplementary material for: Physical activity barriers and facilitators in patients awaiting spine surgery: A qualitative study
Source: PLoS One. 2026 Aug 4;21(8):e0355173. doi: 10.1371/journal.pone.0355173 (PMC13436774; doi:10.1371/journal.pone.0355173)
Supplement: Supplementary file 6 [file pone.0355173.s007.pdf]

Review of PONE-D-26-05753

We thank the Editor and the Reviewers for their thorough evaluation of our manuscript and for the constructive and helpful comments provided. We have carefully considered each suggestion and revised the manuscript accordingly, which we believe has strengthened its clarity and scientific rigor.

Below, we respond to the reviewers' comments point by point. Each comment is reproduced verbatim and labeled as Comment #, followed by our corresponding response labeled as Response #. All changes made to the manuscript have been incorporated into the revised version.

1. Editor:

Journal Requirements

Comment #1. When submitting your revision, we need you to address these additional requirements. Please ensure that your manuscript meets PLOS ONE's style requirements, including those for file naming. The PLOS ONE style templates can be found at https://journals.plos.org/plosone/s/file?id=wjVg/PLOSOne_formatting_sample_main_body.pdf and https://journals.plos.org/plosone/s/file?id=ba62/PLOSOne_formatting_sample_title_authors_affiliations.pdfAuthor

> **Author response #1:** The manuscript has been revised to meet PLOS ONE's style requirements, including file naming conventions, and now fully complies with the journal's guidelines.

Comment #2. We note that the grant information you provided in the 'Funding Information' and 'Financial Disclosure' sections do not match. When you resubmit, please ensure that you provide the correct grant numbers for the awards you received for your study in the 'Funding Information' section.

> **Author response #2:** The mismatch was due to an inconsistency in the funder name: the Financial Disclosure used 'ResearchNB' while the Funding Information listed 'Research New Brunswick'. Both sections now consistently use 'ResearchNB' with grant number IT40020.
> Regarding the grant number, ResearchNB and Mitacs are linked through the same project (IT40020), as ResearchNB served as the partner organization for the Mitacs Accelerate grant. Please note that the Financial Disclosure field was not available for editing during the revision process in Editorial Manager.

Comment #3. Thank you for stating the following financial disclosure: "This work was supported by ResearchNB (https://www.researchnb.ca) and Mitacs (https://www.mitacs.ca) through grant IT40020 awarded to JMG." Please state what role

the funders took in the study. If the funders had no role, please state: "The funders had no role in study design, data collection and analysis, decision to publish, or preparation of the manuscript." If this statement is not correct you must amend it as needed. Please include this amended Role of Funder statement in your cover letter; we will change the online submission form on your behalf.

> **Author response #3:** The funders had no role in study design, data collection and analysis, decision to publish, or preparation of the manuscript. This statement has been added to the cover letter as requested.

Comment #4. We noted in your submission details that a portion of your manuscript may have been presented or published elsewhere. “Yes. An abstract based on this study has been accepted for inclusion at the 26th Annual Scientific Conference of the Canadian Spine Society and will be published in abstract form only. The abstract does not include full methods, detailed results, tables, or figures, and the present submission represents the first full report of the study. Therefore, this does not constitute dual publication.” Please clarify whether this [conference proceeding or publication] was peer-reviewed and formally published. If this work was previously peer-reviewed and published, in the cover letter please provide the reason that this work does not constitute dual publication and should be included in the current manuscript.

> **Author response #4:** The abstract was peer-reviewed and accepted for presentation at a scientific conference, and it will be published in abstract form only. As it contains no detailed methods, results, or figures, this does not constitute dual publication. This has been clarified in the cover letter.

Comment #5. Please note that funding information should not appear in any section or other areas of your manuscript. We will only publish funding information present in the Funding Statement section of the online submission form. Please remove any funding-related text from the manuscript.

> **Author response #5:** The Author Contributions section, including the Funding Acquisition line, has been removed from the manuscript. No funding-related information remains in the manuscript body. All author contributions are entered in the Editorial Manager submission form as required by PLOS ONE.

Comment #6. In the online submission form, you indicated that “Data Availability: Due to the qualitative nature of the study, which includes in-depth interviews containing potentially identifiable and sensitive participant information, the data cannot be made publicly available. De-identified data excerpts supporting the findings are included within the manuscript. Additional data may be made available upon reasonable request to the corresponding author, subject to approval by the relevant ethics committee.”

All PLOS journals now require all data underlying the findings described in their manuscript to be freely available to other researchers, either 1. In a public repository, 2. Within the manuscript itself, or 3. Uploaded as supplementary information.

This policy applies to all data except where public deposition would breach compliance with the protocol approved by your research ethics board. If your data cannot be made publicly available for ethical or legal reasons (e.g., public availability would compromise patient privacy), please explain your reasons on resubmission and your exemption request will be escalated for approval.

> **Author response #6:** We respectfully request an exemption to the data availability policy. The ethics approval granted by the Research Ethics Boards of the Horizon Health Network (2024-3370) and the University of New Brunswick (2024-109) was conditional on ensuring that no published information could be used to identify participants. Given that the primary data underlying this qualitative study consist of verbatim interview transcripts, full public disclosure would be inappropriate and would breach the conditions of our ethics approval, as transcripts may contain details sufficient to identify individual participants despite de-identification efforts.
>
> Beyond the constraints of our ethics approvals, there are well-recognised methodological and ethical considerations regarding the public deposition of qualitative interview transcripts. Qualitative analyses are inherently interpretive and rely on the interaction between researchers and participants, meaning that replication through re-analysis of raw transcripts is not generally considered an appropriate standard for transparency *(Lamb, Russell, Morant, & Stevenson, 2024; Journal of Health Psychology)*. Additionally, even when direct identifiers are removed, qualitative transcripts may contain contextual details that increase the risk of participant re-identification, particularly in studies involving small samples and detailed personal narratives. Recent methodological commentary has therefore argued that public deposition of full qualitative datasets is often neither appropriate nor necessary, and that transparency is better achieved through detailed methodological reporting, reflexivity, coding descriptions, and the inclusion of representative participant quotations *(Lamb, Russell, Morant, & Stevenson, 2024; Journal of Health Psychology).*
>
> In accordance with option 2 of the data availability policy, we have maximised data sharing within the constraints of our ethics approval through two means: (i) de-identified representative quotes are included throughout the Results section of the manuscript, and (ii) a summary table of the number of coded quotes per TDF domain (S1 Table) is provided as supplementary material, offering a quantitative overview of the analytical process. We consider these to represent the maximum level of data that can be shared publicly under the conditions of our ethics approval. Additional data may be made available upon reasonable request to the Horizon Health Network Research Ethics Board (Contact: EthicsServices@HorizonNB.ca) or the University of New Brunswick Research Ethics Board (Contact: ethics@unb.ca).

Comment #7. If the reviewer comments include a recommendation to cite specific previously published works, please review and evaluate these publications to determine whether they are relevant and should be cited. There is no requirement to cite these works unless the editor has indicated otherwise.

> **Author response #7:** No specific references were suggested by the reviewers in their comments.

2. Reviewer #1

Comment #1. This manuscript presents a clinically relevant qualitative study on barriers and facilitators to physical activity among patients awaiting spine surgery using a TDF-informed approach. While the patient-oriented methods and data-prompted interviews are strengths, the analytical depth is limited.

> **Author response #1:** We acknowledge the concern regarding analytical depth and have addressed it through the specific revisions detailed in our responses to Comments #2 through #7 below, which collectively strengthen the methodological transparency, analytical rigor, and interpretative depth of the manuscript.

Abstract

Comment #2. The abstract is well-structured and clearly communicates the study's background, methods, results, and conclusions. However, methodological transparency is limited, particularly regarding how "key domains" were identified and what criteria were used. The term "patient-oriented study" is vague and should be clarified to specify the role of patient involvement. Additionally, "data-prompted interviews" may not be widely understood and would benefit from brief clarification. The conclusion is somewhat generic and should more explicitly state the clinical implications, such as informing tailored preoperative physical activity interventions.

> **Author response #2:** The following changes were made to the abstract:
>
> - Key domains: it is now stated that they *"were determined through researcher consensus based on the frequency and clarity of specific beliefs within each domain."*
> - Patient-oriented study: it is now specified that the patient partner *"contributed to the development of the interview guide and interpretation of findings."*
> - Data-prompted interviews: a clarification has been added: *"(a method in which personalised physical activity information is used to stimulate reflection and discussion)."*
> - Conclusion: clinical implications are now explicitly stated: *"These findings can inform the design of tailored, theory-driven preoperative*

physical activity interventions, targeting key barriers such as pain, fear of harm, and limited access to resources, while leveraging facilitators including motivation, professional guidance, social support, and habit formation."

Introduction

Comment #3. The introduction provides a strong epidemiological and clinical foundation, clearly highlighting the burden of degenerative lumbar conditions and the importance of physical activity. However, the research gap is not sufficiently differentiated from prior qualitative studies, particularly those already exploring patient experiences in similar contexts. The justification for using the TDF is underdeveloped and should be strengthened by explaining its advantages over alternative behavioural models (e.g., COM-B). Furthermore, the study objective would benefit from clearer articulation, explicitly stating that the aim is to identify behavioural determinants using a theory-informed framework.

Author response #3: The following changes were made to the introduction:

- Research gap: the gap has been more clearly differentiated from prior qualitative studies by specifying that, unlike previous research focused on structured exercise-based programs, little is known about patients' broader views on physical activity in everyday life before surgery. A sentence has been added to highlight this distinction (Page 5, Lines 83-85): *"This distinction clinically meaningful, as barriers and facilitators to general physical activity may differ substantially from those encountered in structured, supervised settings."*
- Justification for the TDF: a brief definition of the TDF has been added, along with an explicit justification for its use over broader behavioural models such as the COM-B (Page 5, Lines 89-97): "*The TDF is a validated synthesis of 33 behaviour change theories organised into 12 domains, designed to provide a comprehensive and structured approach to understanding the factors that influence health-related behaviours. Compared to broader behavioural models such as the Capability, Opportunity, Motivation-Behaviour (COM-B) model, the TDF offers greater granularity, enabling a more detailed exploration of the cognitive, emotional, and contextual factors that may influence behaviour. Furthermore, the TDF has been specifically validated for use in qualitative research aimed at identifying barriers and facilitators to behaviour change, and has been applied in patients with musculoskeletal and other chronic conditions."*
- Study objective: the objective has been reformulated to explicitly state that the aim was to identify behavioural determinants using a theory-informed framework (Page 5, Lines 98-100): "*this study aimed to identify potential preoperative behavioural determinants (barriers and facilitators) to*

engagement in physical activity among surgical candidates with lumbar stenosis or lumbar disc herniation, using a theory-informed qualitative framework."

Methodology

Comment #4. The methodology demonstrates several strengths, including ethical rigor, use of COREQ guidelines, and innovative application of data-prompted interviews. However, key aspects require clarification and strengthening. The sampling strategy, described as random, lacks sufficient detail regarding recruitment flow and potential selection bias. The process of data saturation is referenced but not operationally defined, raising concerns about its rigor. The reliance on a single primary coder without reporting inter-coder reliability, reflexivity, or audit procedures introduces potential bias. Additionally, manual coding without qualitative software requires justification. The criteria used to define "key domains," based on "high frequency" and "clear beliefs," remain subjective and insufficiently transparent, limiting reproducibility and methodological robustness.

Author response #4: The following changes were made to Methods:

- Sampling strategy: The sampling strategy has been clarified and expanded in the Patients section. The randomization process is now explicitly described (Page 6, Lines 118-120): "*a randomised contact order was generated using an online randomization tool, and patients were contacted sequentially in that order to avoid selection bias.*" Additionally, the recruitment flow has been moved from the Results section to the Patients section to improve transparency, now reading (Page 6, Lines 120-124): "*Of the 87 patients identified in the registry, 64 met the eligibility criteria, as some had already undergone surgery between registry entry and the time of contact. Of these, 18 consented to participate. The remaining 46 patients did not participate for the following reasons: 23 were not interested in the study, 19 did not respond to the call, and 4 were unable to complete the online interview or could not travel to the interview location.*" The inclusion criteria have also been more clearly articulated in this section.
- Data saturation: The data saturation process has been operationally defined in the Methods section. The iterative process followed is now explicitly described (Pages 10-11, Lines 215-221): "*After an initial set of 10 interviews were conducted and analyzed, three additional interviews were conducted and analyzed to assess whether new barriers or facilitators emerged. If new themes were identified, three further interviews were conducted and analyzed. This iterative process was repeated until no new themes emerged across three consecutive interviews, at which point data saturation was considered reached. Recruitment and analysis were conducted simultaneously to allow*

ongoing saturation assessment. Consensus regarding data saturation was achieved among three researchers (S.D., J.G., J.H.).”

- Single primary coder and inter-coder reliability: the coding process has been clarified and expanded in the Methods section (Pages 11-12, Lines 237-259). It is now explicitly stated that the first two interviews were manually coded by J.G. under the supervision of S.D. to develop a shared coding strategy and resolve initial uncertainties. For the remaining interviews, J.G. consulted S.D. whenever uncertainty arose regarding domain allocation, and consensus was always reached through discussion between the two researchers. Beyond line-by-line coding, the higher-order analytic decisions did not rest with a single researcher. The subthemes drafted by J.G. were reviewed and finalised through two consensus meetings of the wider study team (S.D., J.H., D.F., E.B., and the patient partner A.L.), and the classification of domains as key or other was likewise determined by consensus among these co-investigators. This multi-investigator consensus served as a form of analytic triangulation, further mitigating the risk of single-coder bias.

  The absence of formal inter-rater reliability metrics is justified by the deductive nature of the study and supported by established methodological guidance (Atkins et al., 2017), as coding decisions were systematically guided by the prespecified categories of a predefined TDF. Furthermore, this has been transparently acknowledged as a methodological limitation in the Discussion section.

  The manuscript has been updated as follows
    - In Methods (Page 12, Lines 245-249): *“This supervised approach and ongoing consensus process are consistent with established recommendations for TDF-based qualitative research (Atkins et al., 2017), where coding decisions are guided by a predefined theoretical framework and consensus-based resolution of disagreements is considered an appropriate alternative to independent dual coding (Cofie et al. 2022).”*
    - In Limitations (Page 34, Lines 707-709): *“Finally, formal inter-rater reliability metrics were not calculated. Although coding decisions were guided by the prespecified categories of the TDF framework, the absence of a formal index is acknowledged as a limitation.”*
- Reflexivity: the background of each interviewer is now explicitly described in the Procedure section. D.F. was an undergraduate student researcher with no prior experience in qualitative research but with experience in clinical research involving this patient population, and J.G. was an experienced physiotherapist and post-doctoral researcher with clinical and research expertise in patients with lumbar spine conditions. It is now also stated (Page 9, Lines 178-181): *“prior to data collection, the interviewers were made aware of how their professional backgrounds and*

assumptions might influence the interviews and interpretation of findings, as part of their training with S.D." Additionally, field notes taken during interviews are now described as having been drawn upon during the analytical process to provide additional context.

- Manual coding without qualitative software: We note that the use of qualitative data analysis software for organising and managing data, but does not guarantee analytical rigour. The rigour of qualitative analysis derives from the systematic and transparent application of an analytic framework. Our analysis followed recommendations of TDF based analysis which do not mandate software use (Atkins et al., 2017) using a well-established and validated approach with explicit procedural steps. Coding was conducted line-by-line directly within the manuscript transcripts using close and iterative engagement with the data. We have added the following justification to the manuscript (Page 11, Lines 226-229): *"Coding was conducted line-by-line directly within the interview transcripts. In line with published TDF guidance (Atkins et al., 2017), the use of qualitative data analysis software is not mandated; analytical rigour derives from the systematic and transparent application of the analytic framework rather than the tools used to organise data."*
- Criteria for key domains: The criteria used to identify key domains have been further clarified in the Analysis section. It is now explicitly stated that domain relevance was determined based on two criteria applied specifically to barriers: (i) high frequency of specific beliefs, operationally defined as the comparison of coded quotes across domains, with the full quote counts per domain reported in a new supplementary table (S1 Table); and (ii) presence of clear beliefs, now defined (Page 13, Lines 262-264): *"statements that were explicitly and unambiguously related to physical activity behaviour and expressed consistently across multiple patients."* It is also clarified that facilitators were documented and reported within each key domain but were not used as criteria for determining domain relevance. Finally, we acknowledge that these criteria involve an inherent degree of interpretative judgment, which is consistent with established approaches to TDF-based qualitative research.

Results

Comment #5. The results are comprehensive and well-organised according to TDF domains, supported by rich participant quotations that enhance credibility. However, the section is overly descriptive, with substantial redundancy in both thematic explanations and illustrative quotes. Greater synthesis and higher-order abstraction are needed to improve clarity and readability. Some concepts, particularly pain and functional limitation, are repeated across multiple domains without sufficient integration. The classification of five key domains requires clearer methodological justification.

Additionally, the tables outlining domain criteria (pages 10–11) are informative but overly complex and may be better simplified or moved to supplementary material.

Author response #5: The following changes have been made to the Results section:

- Classification of five key domains: a clarifying sentence has been added at the beginning of the Key domains and other domains section to explain the rationale for classifying domains as key or other (Page 15, Lines 290-293): "*Domains were classified as key or other to prioritise those areas with the strongest perceived influence on physical activity behaviour in this population, which may inform both clinical practice and the development of future interventions. The criteria used for this classification are described in the Methods section and summarised in S4 File.*" Additionally, the Methods section now explicitly describes the criteria used to determine domain relevance, including the frequency and clarity of specific beliefs, as detailed in our response to Comment #4.
- Tables outlining domain criteria: Tables 1 and 2 outlining the domain classification criteria have been moved to supplementary material (S4 File) to improve the readability and flow of the manuscript. References to these tables have been updated accordingly in both the Methods and Results sections.
- Integration of pain and functional limitation across domains: to address the redundancy identified by the reviewer, the descriptions of the five key domains and selected other domains have been revised to explicitly connect the role of pain and functional limitation across domains rather than treating them in isolation. As stated in the introductory paragraph of the key domains section, these themes are described in depth under *beliefs about capabilities*, where they were most prominently expressed, and cross-references have been added throughout the remaining domains to highlight how they interact with each specific domain. In the key domains, for example, physical limitations under *skills* are linked to deconditioning resulting from pain-driven inactivity; *environmental* barriers such as unsafe terrain and adverse weather are connected to patients' balance difficulties and fall-related concerns; and emotional distress under *emotion* is tied to the frustration of being prevented from physical activity by pain and functional limitations, with a bidirectional link to reduced perceived *capability*.

  In the other domains, patients' *motivation* is framed as particularly notable given the physical barriers described *under beliefs about capabilities* and *skills*; *behavioural strategies* are presented as grounded in personal experiential *knowledge* of symptoms rather than formal guidelines; and *daily routines* are linked to *symptom patterns* and *action planning*, reflecting how pain shapes the timing and sustainability of physical activity *habits*.

Discussion

Comment #6. The discussion appropriately contextualises the findings within existing literature and highlights the multifactorial nature of physical activity behaviour in this population. However, much of the section remains descriptive rather than interpretative, with limited exploration of underlying behavioural mechanisms. The introduction of three patient profiles is conceptually interesting but lacks clear methodological grounding and appears speculative. Clinical implications are currently too general and would benefit from more specific, actionable recommendations, such as structured prehabilitation strategies or behaviour-change interventions aligned with TDF domains. While limitations are acknowledged, they should be more critically examined, particularly regarding sample representativeness, lack of data triangulation, and potential researcher bias.

Author response #6: The following changes have been made to the Discussion section:

- Interpretative depth and behavioural mechanisms (Page 31, Lines 643-648): the Principal findings section has been revised to move beyond simple description and offer a deeper interpretation of the psychological and physical experiences underlying the TDF domains. Specifically, we have elaborated on the self-reinforcing cycle of pain, inactivity, and deconditioning. Rather than proposing a causal model, we now describe how these elements coexist. The text clarifies how pain and functional limitations closely connect with diminished beliefs about capabilities, negative beliefs about consequences regarding symptom exacerbation, an erosion of physical skills, and persistent emotional distress.
- Three patient profiles: We acknowledge that the identification of these profiles was not a formal aim of the study, and the text has been revised to address the reviewer's concern about methodological grounding. The profiles are now explicitly framed as observed patterns rather than a systematic classification (Page 36, Lines 746-750): *"The identification of these profiles was not a formal aim of the study. These profiles are not intended as fixed categories but as a heuristic to illustrate population heterogeneity and inform the starting point for individualised clinical assessment, recognising that any intervention must ultimately be tailored to the individual, as discussed below."*
- Clinical implications: the Implications and future research section has been made more specific and actionable. Recommendations are now explicitly aligned with the TDF domains identified as key in this study. A concrete first step is proposed, consisting of (Page 36, Lines 757-759) *"[an] initial conversation [that] would also benefit from addressing symptom-specific concerns, distinguishing between lumbar and leg symptoms, and to explore patients' own views on what kind of support*

would be most helpful." This is followed by more specific recommendations addressing the cycle of pain and inactivity, the role of professional guidance, habit formation, and emotional support, all grounded in the domains identified in this study.

- Limitations: the limitations section has been revised to examine each limitation more critically. The lack of racial and ethnic diversity is now explicitly noted as restricting transferability to more diverse populations (Page 33, Lines 695-696). The absence of data triangulation is acknowledged as limiting the depth and corroboration of the findings, noting that perspectives from healthcare providers or objective activity measures were not included (Page 34, Lines 703-705). Regarding potential researcher bias, this was addressed through explicit reflexivity training, description of interviewers' backgrounds in the Procedure section, and the use of field notes during the analytical process, as described in our response to Comment #4.

Conclusion

Comment #7. The conclusion accurately reflects the study findings and emphasises the interplay of physical, emotional, and contextual factors influencing physical activity. However, it remains somewhat generic and does not sufficiently highlight the study's unique theoretical or clinical contributions. The recommendations for practice and future research are broad and should be made more specific and directly linked to the identified domains. Emphasizing how these findings can inform targeted, theory-driven interventions would significantly enhance the impact of this section.

Author response #7: The following changes have been made to the Conclusion section:

- Theoretical and clinical contributions: the conclusion has been revised to more explicitly highlight the study's unique contributions. The application of the TDF is now foregrounded as the theoretical basis for the findings, and the *"This pattern reveals a self-reinforcing experience where multiple TDF domains coexist: pain and functional limitation co-occurr with diminished beliefs about capabilities, negative beliefs about consequences, an erosion of the physical skills needed to remain physically active, and persistent emotional distress*" is identified as a key conceptual contribution of the study.
- Specific and actionable recommendations: the recommendations for practice have been made more concrete and directly linked to the findings. Rather than broad suggestions, the revised conclusion proposes that *"clinicians should begin with an individualised assessment to explore each patient's barriers, facilitators, beliefs, and symptom-specific concerns, and tailor support accordingly, with particular attention to professional guidance, habit formation, and emotional support"*, all grounded in the domains identified in this study.

- Future research: the recommendations for future research have been refined to emphasise the need for theory-driven interventions informed by the findings of this study, as well as the importance of exploring healthcare providers' perspectives to ensure that strategies are both effective and applicable in practice: *"Future research should test the feasibility of theory-driven interventions informed by these findings and explore the perspectives of healthcare providers to ensure that strategies are both effective and applicable in practice."*

3. Reviewer #2

Comment #1. The manuscript addresses a clinically relevant and underexplored topic, and the use of the Theoretical Domains Framework (TDF) provides a strong conceptual basis. However, the novelty of the study should be emphasised more clearly in the Introduction, particularly in relation to previous qualitative studies involving lumbar spine patients.

> **Author response #1:** We agree that the novelty of the study was not sufficiently emphasised in the original manuscript. In response, we have revised the third paragraph of the Introduction to more explicitly highlight what distinguishes this study from prior qualitative work in lumbar spine patients. Specifically, the manuscript now states (Pages 4-5, Lines 81-85): *"Limited qualitative research has examined the behavioural determinants of general physical activity engagement (as opposed to structured, supervised exercise) in patients awaiting lumbar spine surgery using a theory-informed framework. This distinction is clinically meaningful, as barriers and facilitators to general physical activity may differ substantially from those encountered in structured, supervised settings."* We believe this revision more clearly positions the contribution of the present study within the existing literature.

Comment #2. The study design is appropriate for the research question. Nevertheless, the authors should more explicitly justify why a qualitative approach was chosen over mixed-methods or survey-based approaches, and how this design best captures preoperative physical activity behaviour.

> **Author response #2:** We agree that the rationale for choosing a qualitative design was not sufficiently articulated in the original manuscript. In response, we have added a paragraph at the beginning of the Methods section explicitly justifying the use of a qualitative approach. Specifically, the manuscript now reads (Pages 5-6, Lines 104-108): "*While survey-based approaches can quantify the prevalence of barriers and facilitators, they are constrained by predefined response options and may fail to capture the full range and depth of patients' experiences and perspectives. A qualitative approach, by contrast, allows patients to describe their physical activity behaviour in their own words, revealing*

nuanced cognitive, emotional, and contextual factors that structured instruments are unlikely to detect."
We further note that this is particularly relevant in a surgical population where the lived experience of pain, functional limitation, and uncertainty about surgery is likely to shape physical activity behaviour in ways that cannot be fully anticipated in advance. Finally, we highlight that the use of the TDF as a guiding framework ensured that data were collected and analyzed in a systematic and theoretically grounded manner.

Comment #3. The Methods section is generally well described, but additional details regarding the sampling strategy are needed. Please clarify how many eligible patients were contacted, how many declined participation, and whether any differences existed between participants and non-participants.

Author response #3: Details regarding the sampling strategy, recruitment flow and differences between participants and non-participants have been added to the Patients section, which now reads (Page 6, Lines 118-126): *"A randomised contact order was generated using an online randomization tool, and patients were contacted sequentially in that order to avoid selection bias. Of the 87 patients identified in the registry, 64 met the eligibility criteria, as some had already undergone surgery between registry entry and the time of contact. Of these, 18 consented to participate. The remaining 46 patients did not participate for the following reasons: 23 were not interested in the study, 19 did not respond to the call, and 4 were unable to complete the online interview or could not travel to the interview location. No systematic data were collected on non-participating patients, and therefore a formal comparison between participants and non-participants was not possible."* The inability to formally compare participants and non-participants has also been acknowledged in the limitations section, where we note that it restricts our ability to assess the representativeness of the sample.

Comment #4. Although random sampling from the registry is commendable, the final sample size was relatively small (n=18). The authors should provide a clearer justification for data saturation and explain how saturation was determined in practice.

Author response #4: The process of data saturation paragraph now reads (Pages 10-11, Lines 211-219): *"Although qualitative studies do not require a predetermined sample size, a final sample of 18 patients is consistent with empirical evidence indicating that saturation is typically achieved within 9 to 17 interviews in qualitative studies with relatively homogeneous populations and narrowly defined objectives (Hennink & Kaiser, 2022; Social Science & Medicine). Data saturation was determined following the approach recommended by Francis et al., (Francis et al., 2010; Psychology & Health): after an initial set of 10 interviews were conducted and analyzed, three additional interviews were conducted and analyzed to assess whether new barriers or facilitators emerged.*

If new themes were identified, three further interviews were conducted and analyzed. This iterative process was repeated until no new themes emerged across three consecutive interviews, at which point data saturation was considered reached." Given that our sample exceeds this empirically derived threshold, and that saturation was confirmed through a transparent iterative process with simultaneous recruitment and analysis and consensus among three researchers, we believe the adequacy of the final sample size is well supported.

Comment #5. The role of the patient partner is a strength of the study. Consider expanding how this individual influenced study design, interview guide development, interpretation of findings, and whether they reviewed the final themes.

Author response #5: We have expanded the relevant paragraph in the Interview topic guide section to provide a more comprehensive account of the patient partner's contributions. Specifically, we now clarify that the patient partner (Page 8, Lines 154-160):*"a person with lived experience of degenerative lumbar spine conditions. A.L. contributed to the development of the interview topic guide by providing feedback on the relevance and acceptability of the questions from a patient perspective, participated in the consensus meetings during the analysis phase, reviewed the categorisation of interview extracts into the primary domains confirming their alignment with the patient perspective, reviewed the final themes upon completion of the analysis, and presented the preliminary results at a conference."* We note that the patient partner was not involved in the initial study design, as their engagement began at the stage of interview guide development.

Comment #6. More detail should be provided regarding interviewer reflexivity. Please discuss how the interviewers' professional backgrounds (physiotherapy/research) may have influenced data collection, coding, and interpretation.

Author response #6: We agree that the description of interviewer reflexivity required more detail. The manuscript now reads (Page 9, Lines 181-188): *"J.G.'s background as a physiotherapist with clinical expertise in degenerative lumbar spine conditions may have led to assumptions about the barriers and facilitators patients experience, potentially influencing the formulation of follow-up questions during interviews and the interpretation of response during coding. To mitigate this, reflexivity was actively encouraged throughout the research process: field notes were used to document assumptions during interviews, coding decisions were made under the supervision of S.D. and discussed until consensus was reached, and the use of the TDF as a predefined theoretical framework helped to structure the analysis and limit the influence of subjective interpretation."* We note that reflexivity in the context of coding and interpretation pertains primarily to J.G., as D.F. was involved in data collection but did not conduct the analysis.

Comment #7. The analysis process should be described in greater depth. Clarify whether coding was performed independently by more than one researcher, how disagreements were resolved, and whether qualitative software was used.

Author response #7: The analysis process is now described in the Methods section, and we believe the text now addresses the three points raised:

- Software use: the manuscript now reads (Page 11, Lines 225-229): *"Transcriptions were stored as digital documents and analyzed manually, without the use of software. Coding was conducted line-by-line directly within the interview transcripts. In line with published TDF guidance (Atkins et al. 2017; Implementation Science), the use of qualitative data analysis software is not mandated; analytical rigour derives from the systematic and transparent application of the analytic framework rather than the tools used to organise data."*
- Disagreements: we described in steps 2 and 3 of the Analysis section that coding was conducted under the supervision of S.D. and that all uncertainties were resolved through discussion until consensus was reached. We would further note that disagreement resolution operated at two levels. Coding-level uncertainties were resolved between J.G. and S.D. as described above, while the higher-order analytic decisions, namely the refinement of subthemes and the classification of domains as key or other, were resolved through consensus meetings involving the wider team (S.D., J.H., D.F., E.B., and the patient partner A.L.). Thus, although a single researcher performed the line-by-line coding, the interpretation of the data was subject to multi-investigator consensus.
- Coding: the manuscript now reads (Page 12, Lines 243-249): *"Coding was performed by a single researcher (J.G.). In all cases of uncertainty, consensus was reached through discussion between J.G. and S.D. This supervised approach and ongoing consensus process are consistent with established recommendations for TDF-based qualitative research (Atkins et al. 2017; Implementation Science), where coding decisions are guided by a predefined theoretical framework and consensus-based resolution of disagreements is considered an appropriate alternative to independent dual coding (Cofie et al., 2022; Canadian Medical Education Journal)."*

Comment #8. The decision to identify "key domains" based on frequency and clarity of beliefs should be better justified, as frequency alone does not always reflect importance in qualitative research.

Author response #8: The manuscript now states (Page 13, Lines 264-267): *"Frequency was used alongside belief clarity and consistency, in line with recommendations that domain relevance in TDF-based research should reflect the convergence of multiple indicators rather than quote count alone (Atkins et. al 2017, Implementation Science)."* We would also note that domain relevance

was not judged by a single researcher. The classification of domains as key or other was determined through consensus discussions among the co-investigators (J.G., D.F., S.D., E.B., J.H.) together with the patient partner (A.L.), providing a further safeguard against the limitations of frequency-based judgments.

Comment #9. Results are informative, but the distinction between barriers and facilitators could be presented more clearly, possibly through an additional summary table mapping themes to TDF domains.

Author response #9: The primary organizational principle of this study was the classification of domains into key and other, which reflects the main objective of identifying the domains most likely to influence physical activity behaviour in this population. Within this structure, the distinction between barriers and facilitators is indicated throughout the text using the (b) and (f) labels attached to each illustrative quote, and is explicitly described within each subtheme. To make this clearer, we have added an explicit statement at the beginning of the Results section noting that subthemes are described as barriers, facilitators, or both, reflecting the context-dependent nature of each factor (Page 15, Lines 294-296).

We also considered reorganizing results around a binary barrier/facilitator structure; however, several subthemes function simultaneously as both depending on the patient and context (e.g., guidance and instructions, access to healthcare professionals, weather, daily routines and habits). Imposing such a structure would risk oversimplifying findings that are inherently context-dependent and would not accurately reflect the data.

Comment #10. Consider including a figure or conceptual model summarizing the interaction between pain, emotional distress, environmental constraints, and motivation in shaping physical activity behaviour.

Author response #10: We completely agree that the relationship between pain, emotional distress, environmental constraints, and motivation is highly dynamic and central to understanding these patients' behaviour.

To address this, we have carefully considered how to represent this interplay. Because the TDF is designed as a descriptive framework to identify and map implementation barriers rather than a causal or predictive model, we felt that a visual figure with directional arrows might inadvertently imply strict cause-and-effect relationships that the TDF does not formally mandate. Therefore, instead of a figure, we chose to capture this complex relationship directly in the text. We have updated the Discussion section to explicitly describe how these domains coexist and reinforce one another as a shared, lived experience, avoiding a rigid causal structure while fully exploring how they overlap (Page 31, Lines 645-648): *"This pattern reflects a self-reinforcing experience where multiple TDF domains coexist. Specifically, pain and functional limitation co-*

occurred with diminished beliefs about capabilities, negative beliefs about consequences, an erosion of the physical skills needed to remain physically active, and persistent emotional distress."

Comment #11. The quotations are useful and enhance credibility. However, some quotations may be shortened for readability while preserving meaning.

Author response #11: We have reviewed all quotations and shortened those where meaning could be fully preserved with fewer words, while retaining longer quotes where the full phrasing was necessary to convey emotional depth or contextual nuance. Specifically, we shortened the quotations from Patient 4 (Skills domain), Patient 15 (Beliefs about consequences), Patient 12 (Environmental context and resources), and Patient 4 (Emotion domain). The remaining quotations were kept in their original form, as their length was judged necessary to adequately capture the complexity of the experience described.

Comment #12. The Discussion is strong, but should more clearly distinguish findings specific to lumbar spinal stenosis versus lumbar disc herniation, if relevant differences emerged.

Author response #12: We have reviewed the data carefully to determine whether diagnosis-specific differences in barriers or facilitators emerged between patients with lumbar stenosis and those with lumbar disc herniation. As noted in the Results section, no meaningful differences in perceived capability, avoidance patterns, emotional responses, or contextual factors were identified between the two groups, which is consistent with the chronic and pervasive nature of both conditions.

To address the reviewer's concern more transparently, we have added a sentence in the Principal findings section of the Discussion (Page 31, Lines 640-643): *"No meaningful differences in perceived barriers or facilitators emerged between patients with lumbar stenosis and those with lumbar disc herniation, suggesting that the behavioural mechanisms identified here may apply broadly across degenerative lumbar conditions".*

Additionally, we have added a sentence at the end of the Implications and future research section, which now reads (Page 37, Lines 787-789): *"Future research with larger, diagnosis-specific samples should also examine whether the barriers and facilitators identified here differ between lumbar stenosis and lumbar disc herniation, given that the present study was not powered to detect such differences."*

We believe these additions strengthen the interpretive transparency of the paper without overstating findings that the data do not support.

Comment #13. The authors should expand the clinical implications section by outlining how these findings can be translated into specific prehabilitation or behaviour-change interventions before surgery.

Author response #13: We agree that the clinical implications section would benefit from greater specificity regarding how the findings can be translated into prehabilitation and behaviour-change interventions. We have added a paragraph following the discussion of individualised assessment that maps the key TDF domains identified in this study onto specific behaviour change strategies, which now reads (Pages 36-37, Lines 762-776): *"Beliefs about capabilities and consequences may be addressed through motivational interviewing, which incorporates strategies such as reframing physical activity as safe and beneficial. Emotional barriers suggest the value of acceptance-based strategies to manage fear and frustration. Skills-based limitations support the use of supervised, graded physical activity programs that rebuild confidence through progressive practice. More broadly, intervention should aim to address the self-reinforcing cycle of pain, inactivity, and deconditioning by supporting patients in identifying safe and tolerable activity levels and providing clear, consistent guidance from healthcare professional. Given the prominence of environmental barriers, home-based or hybrid delivery formats should be considered alongside strengthening individual facilitators such as social support. Building and maintaining daily routines and habits appears particularly important, given how difficult patients found it to re-establish activity once disrupted. Structured action planning could support habit formation during the preoperative period, while self-monitoring tools such as activity trackers may help patients during the initial stages before behaviours become automatised."* References which support each sentence are included.

Comment #14. Limitations should be further emphasised, particularly the lack of ethnic diversity, single-center recruitment, possible self-selection bias, and reliance on self-reported physical activity.

Author response #14: We agree that a more robust discussion of the study's limitations strengthens the transparency and interpretation of our findings.
As suggested, we have significantly expanded the Strengths and limitations section, which now reads (Pages 33-34, Lines 694-703): *"First, the study was conducted at a single center, which, combined with the lack of racial and ethnic diversity, may restrict the transferability of the results to different healthcare systems and more diverse populations. The recruitment process may have been subject to self-selection bias, as patients more interested in physical activity might have been more likely to volunteer. The inclusion of two different diagnoses could have introduced variability in experiences, and the imbalance between lumbar stenosis and lumbar disc herniation cases may have influenced the relative weight of perspectives. Reliance on self-reported data also entails a risk of recall or*

response bias, and the absence of objective measures of physical activity, such as accelerometers, means that reported activity levels could not be verified. […].”

Comment #15. The data availability statement may require revision to fully align with PLOS ONE policy. If raw qualitative data cannot be shared due to confidentiality, specify what de-identified excerpts, coding frameworks, or summary datasets can be made available.

Author response #15: We agree with the importance of data transparency and have revised our Data Availability Statement to better align with PLOS ONE policy while strictly maintaining participant confidentiality. While full interview transcripts cannot be shared publicly due to ethical restrictions and the risk of participant identification (REB 2024-3370 / 2024-109), we have provided the following supporting data within the manuscript:

1. De-identified excerpts: Numerous representative quotes are included throughout the Results section to support the themes and findings.
2. S1 Table: We have added a summary table titled “Number of quotes for each domain.” This table provides a quantitative overview of our qualitative coding process, illustrating the distribution of participants' statements across the TDF domains.
3. Institutional Data Access: We have updated the statement to include direct contact information for the relevant Research Ethics Boards for researchers who meet the criteria for access to confidential data, which now reads: *“Due to the sensitive nature of this qualitative study, full interview transcripts cannot be made publicly available as they contain detailed personal accounts that could potentially identify participants, breaching the conditions of our ethics approval (Horizon Health Network REB #2024-3370 and University of New Brunswick REB #2024-109).*
   However, all relevant data supporting the findings are available within the manuscript and its supporting information files:
   - *De-identified representative quotes are included throughout the Results section.*
   - *A summary of the coding framework and the quantitative distribution of quotes per domain is provided in S1 Table.*

   Researchers who meet the criteria for access to confidential data may send requests to the Horizon Health Network Research Ethics Board (Contact: EthicsServices@HorizonNB.ca) or the University of New Brunswick Research Ethics Board (Contact: ethics@unb.ca).”

Comment #16. Please ensure all supplementary files (topic guide, prompt materials, COREQ checklist) are clearly referenced in the manuscript text and available for reviewers/readers.

Author response #16: We have ensured that all supplementary materials are clearly referenced within the manuscript text. Specifically:

- The Interview Topic Guide is referenced as S1 File (Page 7, Line 147).
- The Prompt card materials are referenced as S2 File (Page 8, Line 164).
- The COREQ Checklist is referenced as S3 File (Page 11, Line 223)
- The Number of quotes for each domain is referenced as S1 Table (Page 13, Line 262).
- The Criteria for identifying TDF domains as key is referenced as S4 File (Page 13, Line 271).

Comment #17. Minor language editing is recommended to improve conciseness and remove occasional repetition, particularly in the Discussion section.

Author response #17: We have reviewed the manuscript carefully and made targeted edits to improve conciseness and reduce repetition, particularly in the Results (Other domains) and Discussion (Relation to other studies) sections.
In the other domains subsection, we streamlined the introductory sentences preceding each subtheme, which in several cases restated content already conveyed by the illustrative quotes or by adjacent text.
In the Relation to other studies subsection, we reorganised the content into broader thematic paragraphs, which allowed us to remove repetitive transitional phrases while preserving all substantive comparisons with the existing literature. Throughout this process, we were careful not to remove contextual information that supports the interpretation of findings or that was added in response to previous reviewer comments.
