# Supplementary material for: Physical activity barriers and facilitators in patients awaiting spine surgery: A qualitative study
Source: PLoS One. 2026 Aug 4;21(8):e0355173. doi: 10.1371/journal.pone.0355173 (PMC13436774; doi:10.1371/journal.pone.0355173)
Supplement: Peer review history

# Peer review: Decision Letter 0

**DOI:** 10.1371/journal.pone.0355173.r001

**Authors:** Wanli Zang

24 Apr 2026

PONE-D-26-05753

Physical Activity Barriers and Facilitators in Patients Awaiting Spine Surgery: A Qualitative Study

PLOS One

Dear Dr. García-Moreno,

Thank you for submitting your manuscript to PLOS ONE. After careful consideration, we feel that it has merit but does not fully meet PLOS ONE’s publication criteria as it currently stands. Therefore, we invite you to submit a revised version of the manuscript that addresses the points raised during the review process.

Please submit your revised manuscript by Jun 08 2026 11:59PM. If you will need more time than this to complete your revisions, please reply to this message or contact the journal office at plosone@plos.org. When you're ready to submit your revision, log on to https://www.editorialmanager.com/pone/ and select the 'Submissions Needing Revision' folder to locate your manuscript file.

Please include the following items when submitting your revised manuscript:

- A letter that responds to each point raised by the academic editor and reviewer(s). You should upload this letter as a separate file labeled 'Response to Reviewers'.
- A marked-up copy of your manuscript that highlights changes made to the original version. You should upload this as a separate file labeled 'Revised Manuscript with Track Changes'.
- An unmarked version of your revised paper without tracked changes. You should upload this as a separate file labeled 'Manuscript'.

If you would like to make changes to your financial disclosure, please include your updated statement in your cover letter. Guidelines for resubmitting your figure files are available below the reviewer comments at the end of this letter.

If applicable, we recommend that you deposit your laboratory protocols in protocols.io to enhance the reproducibility of your results. Protocols.io assigns your protocol its own identifier (DOI) so that it can be cited independently in the future. For instructions see: https://journals.plos.org/plosone/s/submission-guidelines#loc-laboratory-protocols. Additionally, PLOS ONE offers an option for publishing peer-reviewed Lab Protocol articles, which describe protocols hosted on protocols.io. Read more information on sharing protocols at https://plos.org/protocols?utm_medium=editorial-email&utm_source=authorletters&utm_campaign=protocols.

As the corresponding author, your ORCID iD is verified in the submission system and will appear in the published article. PLOS supports the use of ORCID, and we encourage all coauthors to register for an ORCID iD and use it as well. Please encourage your coauthors to verify their ORCID iD within the submission system before final acceptance, as unverified ORCID iDs will not appear in the published article. *Only*  the individual author can complete the verification step; PLOS staff *cannot*  verify ORCID iDs on behalf of authors.

We look forward to receiving your revised manuscript.

Kind regards,

Guest Editor

PLOS One

Journal Requirements:

When submitting your revision, we need you to address these additional requirements.

1.Please ensure that your manuscript meets PLOS ONE's style requirements, including those for file naming. The PLOS ONE style templates can be found at

https://journals.plos.org/plosone/s/file?id=wjVg/PLOSOne_formatting_sample_main_body.pdf and

https://journals.plos.org/plosone/s/file?id=ba62/PLOSOne_formatting_sample_title_authors_affiliations.pdf

2. We note that the grant information you provided in the ‘Funding Information’ and ‘Financial Disclosure’ sections do not match.

When you resubmit, please ensure that you provide the correct grant numbers for the awards you received for your study in the ‘Funding Information’ section.

3. Thank you for stating the following financial disclosure:

“This work was supported by ResearchNB (https://www.researchnb.ca) and Mitacs (https://www.mitacs.ca) through grant IT40020 awarded to JMG.”

Please state what role the funders took in the study.  If the funders had no role, please state: "The funders had no role in study design, data collection and analysis, decision to publish, or preparation of the manuscript."

If this statement is not correct you must amend it as needed.

Please include this amended Role of Funder statement in your cover letter; we will change the online submission form on your behalf.

4. We noted in your submission details that a portion of your manuscript may have been presented or published elsewhere. “Yes. An abstract based on this study has been accepted for inclusion at the 26th Annual Scientific Conference of the Canadian Spine Society and will be published in abstract form only. The abstract does not include full methods, detailed results, tables, or figures, and the present submission represents the first full report of the study. Therefore, this does not constitute dual publication.” Please clarify whether this [conference proceeding or publication] was peer-reviewed and formally published. If this work was previously peer-reviewed and published, in the cover letter please provide the reason that this work does not constitute dual publication and should be included in the current manuscript.

5. Please note that funding information should not appear in any section or other areas of your manuscript. We will only publish funding information present in the Funding Statement section of the online submission form. Please remove any funding-related text from the manuscript.

6. In the online submission form, you indicated that “Data Availability: Due to the qualitative nature of the study, which includes in-depth interviews containing potentially identifiable and sensitive participant information, the data cannot be made publicly available. De-identified data excerpts supporting the findings are included within the manuscript. Additional data may be made available upon reasonable request to the corresponding author, subject to approval by the relevant ethics committee.”

All PLOS journals now require all data underlying the findings described in their manuscript to be freely available to other researchers, either 1. In a public repository, 2. Within the manuscript itself, or 3. Uploaded as supplementary information.

This policy applies to all data except where public deposition would breach compliance with the protocol approved by your research ethics board. If your data cannot be made publicly available for ethical or legal reasons (e.g., public availability would compromise patient privacy), please explain your reasons on resubmission and your exemption request will be escalated for approval.

7. If the reviewer comments include a recommendation to cite specific previously published works, please review and evaluate these publications to determine whether they are relevant and should be cited. There is no requirement to cite these works unless the editor has indicated otherwise.

[Note: HTML markup is below. Please do not edit.]

Reviewer's Responses to Questions

**Comments to the Author**

1. Is the manuscript technically sound, and do the data support the conclusions?

The manuscript must describe a technically sound piece of scientific research with data that supports the conclusions. Experiments must have been conducted rigorously, with appropriate controls, replication, and sample sizes. The conclusions must be drawn appropriately based on the data presented.

Reviewer #1: Yes

Reviewer #2: Yes

**********

2. Has the statistical analysis been performed appropriately and rigorously?

Reviewer #1: N/A

Reviewer #2: Yes

**********

3. Have the authors made all data underlying the findings in their manuscript fully available?

The PLOS Data policy requires authors to make all data underlying the findings described in their manuscript fully available without restriction, with rare exception (please refer to the Data Availability Statement in the manuscript PDF file). The data should be provided as part of the manuscript or its supporting information, or deposited to a public repository. For example, in addition to summary statistics, the data points behind means, medians and variance measures should be available. If there are restrictions on publicly sharing data—e.g. participant privacy or use of data from a third party—those must be specified.

Reviewer #1: Yes

Reviewer #2: Yes

**********

4. Is the manuscript presented in an intelligible fashion and written in standard English?

PLOS ONE does not copyedit accepted manuscripts, so the language in submitted articles must be clear, correct, and unambiguous. Any typographical or grammatical errors should be corrected at revision, so please note any specific errors here.

Reviewer #1: Yes

Reviewer #2: Yes

**********

5. Review Comments to the Author

Please use the space provided to explain your answers to the questions above. You may also include additional comments for the author, including concerns about dual publication, research ethics, or publication ethics. (Please upload your review as an attachment if it exceeds 20,000 characters)

Reviewer #1: This manuscript presents a clinically relevant qualitative study on barriers and facilitators to physical activity among patients awaiting spine surgery using a TDF-informed approach. While the patient-oriented methods and data-prompted interviews are strengths, the analytical depth is limited.

ABSTRACT:

The abstract is well-structured and clearly communicates the study’s background, methods, results, and conclusions. However, methodological transparency is limited, particularly regarding how “key domains” were identified and what criteria were used. The term “patient-oriented study” is vague and should be clarified to specify the role of patient involvement. Additionally, “data-prompted interviews” may not be widely understood and would benefit from brief clarification. The conclusion is somewhat generic and should more explicitly state the clinical implications, such as informing tailored preoperative physical activity interventions.

INTRODUCTION:

The introduction provides a strong epidemiological and clinical foundation, clearly highlighting the burden of degenerative lumbar conditions and the importance of physical activity. However, the research gap is not sufficiently differentiated from prior qualitative studies, particularly those already exploring patient experiences in similar contexts. The justification for using the TDF is underdeveloped and should be strengthened by explaining its advantages over alternative behavioral models (e.g., COM-B). Furthermore, the study objective would benefit from clearer articulation, explicitly stating that the aim is to identify behavioral determinants using a theory-informed framework.

METHODOLOGY:

The methodology demonstrates several strengths, including ethical rigor, use of COREQ guidelines, and innovative application of data-prompted interviews. However, key aspects require clarification and strengthening. The sampling strategy, described as random, lacks sufficient detail regarding recruitment flow and potential selection bias. The process of data saturation is referenced but not operationally defined, raising concerns about its rigor. The reliance on a single primary coder without reporting inter-coder reliability, reflexivity, or audit procedures introduces potential bias. Additionally, manual coding without qualitative software requires justification. The criteria used to define “key domains,” based on “high frequency” and “clear beliefs,” remain subjective and insufficiently transparent, limiting reproducibility and methodological robustness.

RESULTS:

The results are comprehensive and well-organized according to TDF domains, supported by rich participant quotations that enhance credibility. However, the section is overly descriptive, with substantial redundancy in both thematic explanations and illustrative quotes. Greater synthesis and higher-order abstraction are needed to improve clarity and readability. Some concepts, particularly pain and functional limitation, are repeated across multiple domains without sufficient integration. The classification of five key domains requires clearer methodological justification. Additionally, the tables outlining domain criteria (pages 10–11) are informative but overly complex and may be better simplified or moved to supplementary material.

DISCUSSION:

The discussion appropriately contextualizes the findings within existing literature and highlights the multifactorial nature of physical activity behavior in this population. However, much of the section remains descriptive rather than interpretative, with limited exploration of underlying behavioral mechanisms. The introduction of three patient profiles is conceptually interesting but lacks clear methodological grounding and appears speculative. Clinical implications are currently too general and would benefit from more specific, actionable recommendations, such as structured prehabilitation strategies or behavior-change interventions aligned with TDF domains. While limitations are acknowledged, they should be more critically examined, particularly regarding sample representativeness, lack of data triangulation, and potential researcher bias.

CONCLUSION:

The conclusion accurately reflects the study findings and emphasizes the interplay of physical, emotional, and contextual factors influencing physical activity. However, it remains somewhat generic and does not sufficiently highlight the study’s unique theoretical or clinical contributions. The recommendations for practice and future research are broad and should be made more specific and directly linked to the identified domains. Emphasizing how these findings can inform targeted, theory-driven interventions would significantly enhance the impact of this section.

Reviewer #2: 1 - The manuscript addresses a clinically relevant and underexplored topic, and the use of the Theoretical Domains Framework (TDF) provides a strong conceptual basis. However, the novelty of the study should be emphasized more clearly in the Introduction, particularly in relation to previous qualitative studies involving lumbar spine patients.

2 - The study design is appropriate for the research question. Nevertheless, the authors should more explicitly justify why a qualitative approach was chosen over mixed-methods or survey-based approaches, and how this design best captures preoperative physical activity behavior.

3 - The Methods section is generally well described, but additional details regarding the sampling strategy are needed. Please clarify how many eligible patients were contacted, how many declined participation, and whether any differences existed between participants and non-participants.

4 - Although random sampling from the registry is commendable, the final sample size was relatively small (n=18). The authors should provide a clearer justification for data saturation and explain how saturation was determined in practice.

5 - The role of the patient partner is a strength of the study. Consider expanding how this individual influenced study design, interview guide development, interpretation of findings, and whether they reviewed the final themes.

6 - More detail should be provided regarding interviewer reflexivity. Please discuss how the interviewers’ professional backgrounds (physiotherapy/research) may have influenced data collection, coding, and interpretation.

7 - The analysis process should be described in greater depth. Clarify whether coding was performed independently by more than one researcher, how disagreements were resolved, and whether qualitative software was used.

8 - The decision to identify “key domains” based on frequency and clarity of beliefs should be better justified, as frequency alone does not always reflect importance in qualitative research.

9 - Results are informative, but the distinction between barriers and facilitators could be presented more clearly, possibly through an additional summary table mapping themes to TDF domains.

10 - Consider including a figure or conceptual model summarizing the interaction between pain, emotional distress, environmental constraints, and motivation in shaping physical activity behavior.

11 - The quotations are useful and enhance credibility. However, some quotations may be shortened for readability while preserving meaning.

12 - The Discussion is strong, but should more clearly distinguish findings specific to lumbar spinal stenosis versus lumbar disc herniation, if relevant differences emerged.

13 - The authors should expand the clinical implications section by outlining how these findings can be translated into specific prehabilitation or behavior-change interventions before surgery.

14 - Limitations should be further emphasized, particularly the lack of ethnic diversity, single-center recruitment, possible self-selection bias, and reliance on self-reported physical activity.

15 - The data availability statement may require revision to fully align with PLOS ONE policy. If raw qualitative data cannot be shared due to confidentiality, specify what de-identified excerpts, coding frameworks, or summary datasets can be made available.

16 - Please ensure all supplementary files (topic guide, prompt materials, COREQ checklist) are clearly referenced in the manuscript text and available for reviewers/readers.

17 - Minor language editing is recommended to improve conciseness and remove occasional repetition, particularly in the Discussion section.

**********

6. PLOS authors have the option to publish the peer review history of their article (what does this mean?). If published, this will include your full peer review and any attached files.

If you choose “no”, your identity will remain anonymous but your review may still be made public.

**Do you want your identity to be public for this peer review?**  For information about this choice, including consent withdrawal, please see our Privacy Policy.

Reviewer #1: No

Reviewer #2: No

**********

[NOTE: If reviewer comments were submitted as an attachment file, they will be attached to this email and accessible via the submission site. Please log into your account, locate the manuscript record, and check for the action link "View Attachments". If this link does not appear, there are no attachment files.]

To ensure your figures meet our technical requirements, please review our figure guidelines: https://journals.plos.org/plosone/s/figures

You may also use PLOS’s free figure tool, NAAS, to help you prepare publication quality figures: https://journals.plos.org/plosone/s/figures#loc-tools-for-figure-preparation.

NAAS will assess whether your figures meet our technical requirements by comparing each figure against our figure specifications.

# Author comment: Author response to Decision Letter 1

**DOI:** 10.1371/journal.pone.0355173.r002

8 Jul 2026

We thank the Editor and Reviewers for their valuable comments on our manuscript. We have carefully revised the manuscript accordingly and uploaded a "Response to Reviewers" document, in which we address each comment from the Editor and Reviewers point by point and detail the corresponding changes made to the manuscript.

We have also updated the Data Availability Statement as requested.

Thank you for your consideration.

Attachment Submitted filename: Response to Reviewers.pdf

# Peer review: Decision Letter 1

**DOI:** 10.1371/journal.pone.0355173.r003

**Authors:** Wanli Zang

20 Jul 2026

Physical Activity Barriers and Facilitators in Patients Awaiting Spine Surgery: A Qualitative Study

PONE-D-26-05753R1

Dear Dr. García-Moreno,

We’re pleased to inform you that your manuscript has been judged scientifically suitable for publication and will be formally accepted for publication once it meets all outstanding technical requirements.

Within one week, you’ll receive an e-mail detailing the required amendments. When these have been addressed, you’ll receive a formal acceptance letter and your manuscript will be scheduled for publication.

An invoice will be generated when your article is formally accepted. Please note, if your institution has a publishing partnership with PLOS and your article meets the relevant criteria, all or part of your publication costs will be covered. Please make sure your user information is up-to-date by logging into Editorial Manager at Editorial Manager® and clicking the ‘Update My Information' link at the top of the page. For questions related to billing, please contact billing support.

If your institution or institutions have a press office, please notify them about your upcoming paper to help maximize its impact. If they’ll be preparing press materials, please inform our press team as soon as possible -- no later than 48 hours after receiving the formal acceptance. Your manuscript will remain under strict press embargo until 2 pm Eastern Time on the date of publication. For more information, please contact onepress@plos.org.

Kind regards,

Wanli Zang, Ph.D.

Guest Editor

PLOS One

Additional Editor Comments (optional):

Reviewers' comments:

Reviewer's Responses to Questions

**Comments to the Author**

1. If the authors have adequately addressed your comments raised in a previous round of review and you feel that this manuscript is now acceptable for publication, you may indicate that here to bypass the “Comments to the Author” section, enter your conflict of interest statement in the “Confidential to Editor” section, and submit your "Accept" recommendation.

Reviewer #1: All comments have been addressed

Reviewer #2: All comments have been addressed

**********

2. Is the manuscript technically sound, and do the data support the conclusions?

The manuscript must describe a technically sound piece of scientific research with data that supports the conclusions. Experiments must have been conducted rigorously, with appropriate controls, replication, and sample sizes. The conclusions must be drawn appropriately based on the data presented.

Reviewer #1: Yes

Reviewer #2: Yes

**********

3. Has the statistical analysis been performed appropriately and rigorously?

Reviewer #1: N/A

Reviewer #2: Yes

**********

4. Have the authors made all data underlying the findings in their manuscript fully available?

The PLOS Data policy requires authors to make all data underlying the findings described in their manuscript fully available without restriction, with rare exception (please refer to the Data Availability Statement in the manuscript PDF file). The data should be provided as part of the manuscript or its supporting information, or deposited to a public repository. For example, in addition to summary statistics, the data points behind means, medians and variance measures should be available. If there are restrictions on publicly sharing data—e.g. participant privacy or use of data from a third party—those must be specified.

Reviewer #1: Yes

Reviewer #2: Yes

**********

5. Is the manuscript presented in an intelligible fashion and written in standard English?

PLOS ONE does not copyedit accepted manuscripts, so the language in submitted articles must be clear, correct, and unambiguous. Any typographical or grammatical errors should be corrected at revision, so please note any specific errors here.

Reviewer #1: Yes

Reviewer #2: Yes

**********

6. Review Comments to the Author

Please use the space provided to explain your answers to the questions above. You may also include additional comments for the author, including concerns about dual publication, research ethics, or publication ethics. (Please upload your review as an attachment if it exceeds 20,000 characters)

Reviewer #1: Based on the revised manuscript and the authors' responses, the authors have addressed nearly all of the substantive concerns raised during the previous review. The revisions have considerably improved the methodological transparency, strengthened the theoretical justification, clarified the analytical procedures, and enhanced the discussion of the study's clinical implications.

1. ABSTRACT

The authors have satisfactorily addressed my comments regarding the abstract. The methodological description is now clearer, particularly the explanation of the patient-oriented approach, the definition of data-prompted interviews, and the identification of key domains. The authors have also strengthened the conclusion by emphasizing theory-driven and preoperative interventions.

2. INTRODUCTION

The authors have adequately addressed my comments concerning the introduction. The research gap is now more clearly articulated, the rationale for selecting the TDF over broader behavioral models has been strengthened, and the study objective is explicitly aligned with a theory-informed investigation of behavioral determinants. The Introduction now provides a stronger conceptual foundation for the study.

3. METHODOLOGY

The authors have substantially addressed my methodological concerns. The revised manuscript now provides a much clearer description of participant recruitment, sampling procedures, operational criteria for data saturation, reflexivity, coding procedures, and the rationale for manual analysis. Although formal inter-coder reliability was not incorporated, the authors have provided an appropriate justification based on established TDF methodological guidance and consensus coding procedures. Overall, the methodological transparency has been considerably improved, and I consider this issue satisfactorily addressed.

4. RESULTS

The results section now integrates recurring themes more effectively and provides a clearer explanation of the criteria used to identify key domains.

5. DISCUSSION

The discussion has been substantially strengthened in response to my comments. The authors provide a more interpretative discussion of the findings, expand the clinical implications by linking behavioral determinants to potential intervention strategies, and more critically acknowledge methodological limitations. Although the proposed patient profiles remain somewhat conceptual, they are now presented with appropriate caution and do not detract from the overall quality of the discussion.

6. CONCLUSION

The authors have satisfactorily revised the conclusion. The section now more clearly emphasizes the theoretical contribution of the study and provides more specific recommendations for future practice and research based on the identified behavioral determinants.

Reviewer #2: (No Response)

**********

7. PLOS authors have the option to publish the peer review history of their article (what does this mean?). If published, this will include your full peer review and any attached files.

If you choose “no”, your identity will remain anonymous but your review may still be made public.

**Do you want your identity to be public for this peer review?**  For information about this choice, including consent withdrawal, please see our Privacy Policy.

Reviewer #1: No

Reviewer #2: No

**********

# Editor report: Acceptance letter

**DOI:** 10.1371/journal.pone.0355173.r004

**Authors:** Wanli Zang

PONE-D-26-05753R1

PLOS One

Dear Dr. García-Moreno,

I'm pleased to inform you that your manuscript has been deemed suitable for publication in PLOS One. Congratulations! Your manuscript is now being handed over to our production team.

At this stage, our production department will prepare your paper for publication. This includes ensuring the following:

* All references, tables, and figures are properly cited

* All relevant supporting information is included in the manuscript submission,

* There are no issues that prevent the paper from being properly typeset

You will receive further instructions from the production team, including instructions on how to review your proof when it is ready. Please keep in mind that we are working through a large volume of accepted articles, so please give us a few days to review your paper and let you know the next and final steps.

Lastly, if your institution or institutions have a press office, please let them know about your upcoming paper now to help maximize its impact. If they'll be preparing press materials, please inform our press team within the next 48 hours. Your manuscript will remain under strict press embargo until 2 pm Eastern Time on the date of publication. For more information, please contact onepress@plos.org.

You will receive an invoice from PLOS for your publication fee after your manuscript has reached the completed accept phase. If you receive an email requesting payment before acceptance or for any other service, this may be a phishing scheme. Learn how to identify phishing emails and protect your accounts at https://explore.plos.org/phishing.

If we can help with anything else, please email us at customercare@plos.org.

Thank you for submitting your work to PLOS ONE and supporting open access.

Kind regards,

PLOS ONE Editorial Office Staff

on behalf of

Dr. Wanli Zang

Guest Editor

PLOS One
